# KSR1 is a scaffold for the Hippo signaling pathway

**DOI:** 10.1038/s42003-025-09009-4

**Published:** 2025-12-01

**Authors:** Samar Sayedyahossein, Mohammed Rizwan Babu Sait, Zhigang Li, Riddhi Banerjee, Andy Tran, Louise Thines, Mehdi Karimi, Mehrnoosh Bahmani, Naomi Mishan, Pooya Borzou, Sergio A. Hassan, David B. Sacks

**Affiliations:** 1 Department of Laboratory Medicine, National Institutes of Health, Bethesda, MD USA; 2 Confocal Microscopy Core Facility, Laboratory of Cancer Biology and Genetics, NCI, National Institutes of Health, Bethesda, MD USA; 3 Department of Mathematics, Illinois State University, Normal, IL USA; 4 Department of Physics and Chemistry, Illinois State University, Normal, IL USA; 5 Department of Physiology and Pharmacology, University of Western Ontario, London, ON Canada; 6 Department of Electrical and Computer Engineering, Isfahan University of Technology, Isfahan, Iran; 7 Bioinformatics and Computational Biosciences Branch, OCICB, National Institute of Allergy and Infectious Diseases (NIAID), National Institutes of Health, Bethesda, MD USA

**Keywords:** Cell signalling, Pathogenesis

## Abstract

The evolutionarily conserved Hippo signaling pathway regulates organ size and tissue homeostasis. Yes-associated protein (YAP) functions as a transcriptional co-activator and is a critical downstream effector of the Hippo signaling pathway. Altered crosstalk with oncogenic signaling pathways contributes to YAP dysregulation in cancer. Kinase Suppressor of Ras 1 (KSR1) scaffolds the Ras cascade. Some of the functions of the Ras and Hippo pathways in regulating cellular processes are similar. Nevertheless, the potential intersection of Ras and Hippo signaling has not been explored. Here, we identify KSR1 as a previously unrecognized scaffold of the Hippo pathway. We demonstrate that KSR1 constitutively binds to YAP and MST1 and forms a complex with LATS1. Moreover, KSR1 modulates YAP protein levels and its transcriptional activity, at least in part through the RhoA/actin axis. Our findings provide insight into the role of KSR1 as a scaffold of the Hippo signaling that could yield novel therapeutics.

## Abstract (web summary)

KSR1 interacts with and regulates YAP co-transcriptional function, revealing an additional mechanism for growth control and tumourigenesis.

## Introduction

The evolutionarily conserved Hippo pathway plays a pivotal role in regulating fundamental cellular processes, including proliferation, differentiation, and apoptosis^1^. Hippo is instrumental in organ development, maintaining tissue homeostasis, and facilitating regeneration^2^. Dysregulation of Hippo signaling promotes tumor initiation, progression, and resistance to treatment of various types of cancers^3^. The Hippo pathway does not rely on a dedicated receptor; instead, it integrates signals from a wide array of receptors and stimuli, both from within the cell and its environment^1^. This allows Hippo signaling to respond to diverse cellular conditions, orchestrating several biological outcomes. The core kinases of the Hippo pathway are mammalian sterile 20-like protein kinase 1 and 2 (MST1, MST2) and large tumor suppressor kinase 1 and 2 (LATS1, LATS2). When Hippo signaling is “ON”, MST1/2 bind to and phosphorylates LATS1/2. Once activated, LATS1/2 phosphorylate and sequester in the cytoplasm the transcriptional co-activators, Yes-associated protein (YAP) and transcriptional co-activator with PDZ-binding motif (TAZ). When Hippo is “OFF”, unphosphorylated YAP/TAZ translocate to the nucleus where they bind to the TEAD family of transcription factors and control transcription of genes involved in proliferation and survival^2^. Increased expression and/or enhanced nuclear localization of YAP/TAZ is implicated in tumorigenesis and poor prognosis in numerous cancers^3^, including, but not limited to, lung carcinoma^4^, colorectal carcinoma^5^, skin squamous cell carcinoma^6^ and invasive breast carcinoma^7^.

YAP activity is regulated by several intracellular and extracellular stimuli, such as Ca^2+^ signaling, mechanical cues and actin remodeling^8^. Actin polymerization in response to increased active RhoA, a small GTPase, is a priming signal that enhances YAP nuclear localization and increases the expression of YAP target genes^9^, including cysteine-rich angiogenic inducer 61 (CyR61) and connective tissue growth factor (CTGF)^10^.

The Ras-Raf-MEK (mitogen-activated protein kinase kinase)-ERK (extracellular signal-regulated kinase) signaling pathway comprises a series of conserved molecular cascades that orchestrate fundamental cellular activities, including proliferation, differentiation and response to stress signals^11^. The highly conserved scaffold protein, kinase suppressor of Ras1 (KSR1)^12^, coordinates the assembly of Raf–MEK–ERK into functional complexes^13^ and acts as a regulatory hub, modulating cellular reactions. Mice lacking *ksr1* are phenotypically normal, although optimal mitogen-activated protein kinase (MAPK) pathway activation and transformation by Ras oncogenes are attenuated^14,15^. In addition to the main components of the Ras-Raf-MEK-ERK network, KSR1 binds directly to several intracellular proteins, such as calmodulin^16^, IQGAP1^17^, 14-3-3^18^, G protein-βγ subunit^19^, chaperones HSP70, HSP90, Cdc37^20^ and Cdc25C-associated kinase 1 (C-TAK1 kinase)^21^. The stability and subcellular localization of KSR1 are influenced by its interactions with binding partners, such as C-TAK1^21^.

While both the Ras cascade and the Hippo pathway individually influence tissue homeostasis, the potential crosstalk between these pathways remains unexplored. Therefore, in this study, we addressed the hypothesis that the Ras and Hippo cascades intersect, and investigated whether KSR1 mediates crosstalk between Ras and Hippo signaling.

Here, we show that KSR1 scaffolds core components of the Hippo pathway. KSR1 binds directly to MST1 and YAP in vitro and forms a complex with MST1, LATS1 and YAP in cells. Importantly, the formation of KSR1, MST1, LATS1 and YAP complexes is modulated by epidermal growth factor (EGF). In addition, KSR1 regulates YAP mRNA levels as well as YAP protein subcellular localization, in part through RhoA/actin. Overall, our data identify KSR1 as a previously unrecognized scaffold of the Hippo pathway that positively regulates YAP function through an MST1, RhoA, actin network.

## Results

### KSR1 binds to MST1 and LATS1, core kinases of the Hippo pathway

**Fig. 1:**
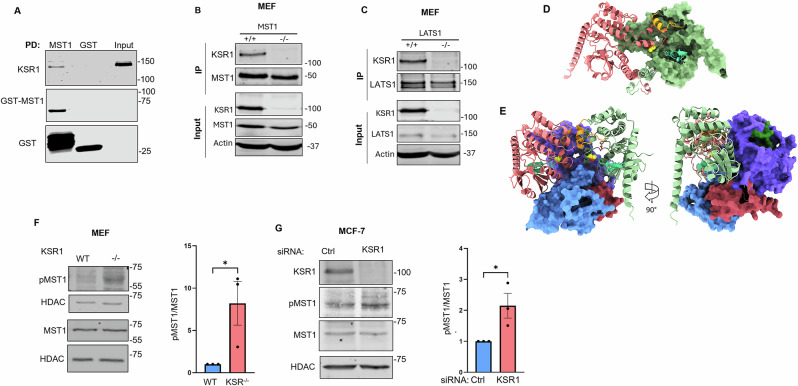
KSR1 binds to and regulates the core kinases of the Hippo pathway. **A** KSR1, generated by T_N_T, was incubated with GST-MST1 or GST alone, both expressed in *Escherichia coli*. GST pull-down (PD) with glutathione-Sepharose was conducted. Samples were resolved by SDS-PAGE and Western blotting. Blots were probed with antibodies against KSR1 and GST. The positions of migration of molecular weight markers are indicated on the right. **B**, **C** KSR1^+/+^ (+/+) and KSR1^−/−^ (–/–) MEFs were lysed and immunoprecipitated (IP) with anti-MST1 (**B**) or anti-LATS1 (**C**) antibodies. Samples were analyzed by Western blotting. All inputs in **A**–**C** represent 5% of sample volume. **D**, **E** Computational models of MST1_2_ (**D**) and KSR1:MST1_2_ (**E**) with important structural motifs highlighted. **D** MST1 monomers (UniProt AC: Q13043) are colored green and pink. The αG helix (residues 220-240), amber, the active site (proton acceptor D149), red, and the ATP-binding site (33-44, 59), aqua, are shown in one of the monomers (green) with their corresponding molecular surfaces removed. The activation loop (residues 170-190, white arrow) of the other monomer (pink) contains the phosphorylation site Thr^183^ (spherical atoms). **E** KSR1 (UniProt AC: QIVT5) is shown in a molecular surface representation, with the N- (30-190) and C- (595-885) terminal domains colored blue and purple, respectively. The Zn-binding domain is colored wine, and the catalytic domain, green. MST1 monomers are colored as in (**D**). **F** Equal amounts of protein lysate from serum starved WT and KSR1^–/–^ MEFs were resolved by Western blotting, probed with antibodies to phosphorylated MST1 (pMST1) and total MST1. HDAC was loading control. Bands were quantified with Image Studio 2.0 (LI-COR Biosciences). pMST1 expression was corrected for total MST1 in the corresponding sample. Data are expressed as means ±S.E. (error bars), with WT MEFs set as 1 (*N* = 3). **G** MCF-7 cells, transfected with control (Ctrl) or KSR1 specific siRNA, were lysed, resolved by Western blotting and probed with antibodies to KSR1, pMST1, MST1, and HDAC. pMST1/MST1 was quantified (*N* = 3). *p < 0.05, Student’s *t* test. All blots are representative of ≥3 independent experiments.

The MAPK scaffold protein IQGAP1^22^ also scaffolds Hippo^23,24^. To determine whether KSR1 serves a scaffold in the Hippo pathway, we initially evaluated whether KSR1 interacts with the Hippo kinase, MST1, in vitro. Glutathione S-transferase (GST)-tagged MST1, expressed in *Escherichia coli*, was incubated with KSR1, produced by an in vitro transcription and translation (T_N_T) system. GST pull-downs were processed by SDS-PAGE and Western blotting, which showed that KSR1 binds to MST1 (Fig. 1A). GST-Sepharose alone did not bind KSR1. These data demonstrate that KSR1 binds MST1 in vitro.

To ascertain whether KSR1 and MST1 bind in a normal cell milieu, we immunoprecipitated MST1 from mouse embryonic fibroblasts (MEFs). Analysis revealed that KSR1 co-immunoprecipitated with MST1 from MEFs that stably overexpress KSR1 (KSR1^+/+^ MEFs) (Fig. 1B). As anticipated, no KSR1 was detected in immunoprecipitates from KSR1-null MEFs (KSR1^–/–^ MEFs) (Fig. 1B). Next, we evaluated the association between endogenous KSR1 and MST1 in MCF-7 breast carcinoma cells. Consistent with our observations in KSR1^+/+^ MEFs, endogenous KSR1 immunoprecipitated with endogenous MST1 from MCF-7 cells (Supplementary Fig. 1A). Moreover, neither KSR1 nor MST1 was present in samples precipitated with non-specific antibodies against IgG. Taken together, these data reveal that KSR1 binds directly to MST1 in vitro and forms a complex with MST1 in cells.

Scaffold proteins assemble signaling complexes^25^. To evaluate whether KSR1 is a scaffold in the Hippo pathway, we examined whether it binds to LATS1, the kinase immediately downstream of MST1. Immunoprecipitation of endogenous LATS1 revealed co-immunoprecipitation of KSR1 from KSR1^+/+^ MEFs (Fig. 1C). No KSR1 was detected in immunoprecipitates from KSR1^–/–^ MEFs. To further validate the interaction between KSR1 and LATS1 in a different cell line, we transfected HEK293T cells with GFP-tagged KSR1. Immunofluorescent analysis and confocal microscopy revealed that KSR1 co-localizes with endogenous LATS1 in cells (Supplementary Fig. 1B). To investigate whether the association between KSR1 and LATS1 is direct, we conducted mass photometry using pure KSR1 and LATS1 proteins (Supplementary Fig. 1C). The combined sample of KSR1 and LATS1 proteins exhibited a distinct shift to a higher molecular mass than the individual proteins, indicative of formation of a higher-order complex. These findings support a direct, in vitro interaction between KSR1 and LATS1. Collectively, our data demonstrate that KSR1 binds both MST1 and LATS1, kinases in the Hippo pathway.

### Computational modeling with AlphaFold2

Activation of MST1 relies on trans-autophosphorylation on Thr^183^, which is mediated by dimerization^26^. To gain structural insight, we used AlphaFold2^27^ multimer to create models of the MST1 dimer, termed MST1_2_, and of MST1_2_:KSR1 complexes (Supplementary Fig. 1D). The best model of MST1_2_ shows that the activation loop of one monomer, containing the activating phosphorylation site Thr^183^, extends into the cavity formed by the αG helix and the catalytic site of its partner (Fig. 1D and Supplementary Fig. 1), suggesting trans-autophosphorylation. In the KSR1:MST1_2_ complex, the MST1 monomers dock into the wedge formed by the N- and C-terminal domains of KSR1 and have similar relative orientations. However, the activation loops retract, and the catalytic sites become partially occluded and perturbed by KSR1 (Fig. 1E). The computational model suggests that KSR1 binding would inhibit MST1 autophosphorylation.

To test this hypothesis experimentally, we examined the effect of KSR1 on phosphorylation of MST1 on amino acid Thr^183^, which reflects Hippo activation^28^. We compared the effects of serum starvation, which activates Hippo^29^, on MST1 phosphorylation in control cells to that in KSR1-null cells. The data show that the amount of phosphorylated MST1 in KSR1^−/−^ MEFs is significantly greater than in control cells (Fig. 1F). Consistent with this observation, knocking down KSR1 by siRNA in MCF-7 human breast carcinoma cells significantly increases by 1.8-fold the amount of phosphorylated MST1 (Fig. 1G). Taken together with the structural modeling, these results imply that KSR1 binding may inhibit MST1 activation.

### KSR1 binds to YAP in vitro and in cells

**Fig. 2:**
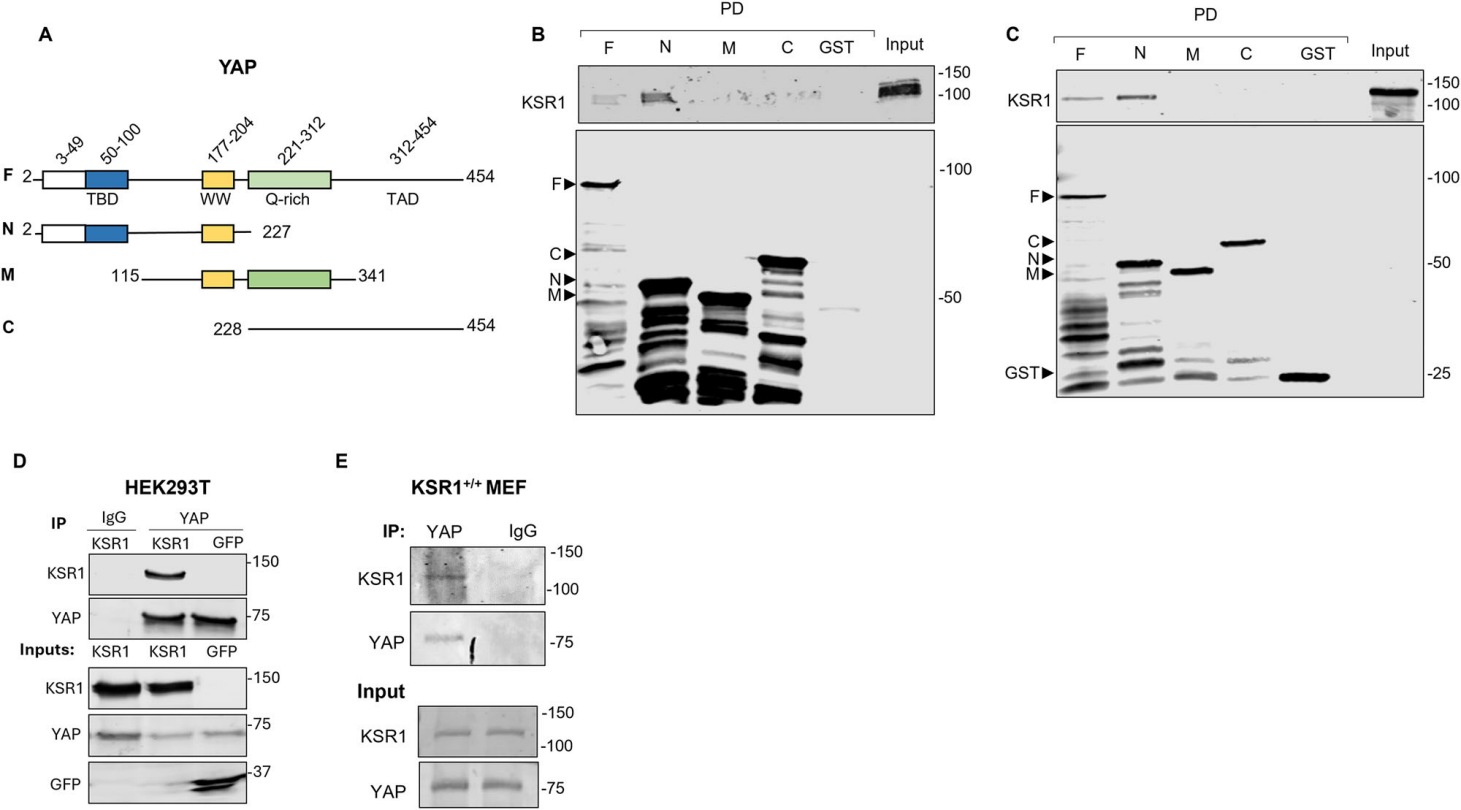
KSR1 binds to YAP in vitro and in cells. **A** Schematic representation of YAP constructs. Full-length (F) and fragments, N (amino acids 2-227), M (aa 115-341) and C (aa 228-454) of YAP are depicted. TEAD-binding domain (TBD), tryptophan-containing domain (WW) and transcription activation domain (TAD). **B** KSR1, generated by T_N_T, was incubated with equal amounts of each GST-tagged YAP construct. GST-Sepharose was the negative control. GST pull-down (PD) was performed and samples were resolved by Western blotting. Blots were probed with anti-KSR1 (upper panel) and anti-GST (lower panel) antibodies. **C** KSR1^+/+^ MEF lysates were incubated with the GST-YAP fragments. Samples were processed as described for (**B**). **D** HEK293T cells were transfected with GFP alone or GFP-tagged KSR1 (KSR1). After 72 h, cells were lysed and immunoprecipitated with either anti-YAP antibodies or rabbit IgG (negative control). Whole cell lysates were processed in parallel. **E** KSR1^+/+^ MEFs were lysed and immunoprecipitated with anti-YAP antibodies. Rabbit IgG was negative control. Samples were processed as described for (**D**). All inputs in **B**–**E** represent 5% of the samples. All data in this figure are representative of at least 3 independent experiments.

KSR1 scaffolding enables signal propagation in the MAPK pathway, with the extent of ERK phosphorylation routinely used as a readout of pathway activation^30^. In Hippo signaling, a complex of MST1, LATS1 and YAP modulates the phosphorylation of YAP^31^. Binding of KSR1 to both MST1 and LATS1 raised the question of whether KSR1 also interacts with YAP. We tested this hypothesis by two approaches. To ascertain whether these proteins associate directly, we generated GST-tagged YAP (Fig. 2A), expressed it in *E. coli* and incubated it with KSR1 produced by T_N_T. Western blotting of GST pull-downs revealed that KSR1 binds to full-length YAP (Fig. 2B). To narrow the binding region, we generated shorter fragments of YAP (Fig. 2A) and performed similar experiments. Analysis revealed that KSR1 binds to the N-terminal region of YAP, but not to the C-terminal or middle portions (Fig. 2B). Binding was specific as no KSR1 was detected in samples incubated with GST-Sepharose. In a complementary approach, we incubated lysates obtained from KSR1^+/+^ MEFs with GST-YAP. Analogous to the findings with purified proteins, KSR1 binds only to full-length YAP and its N-terminal half (Fig. 2C). Our results show that KSR1 binds directly to YAP between amino acids 2 and 227.

Having established direct binding between KSR1 and YAP, we evaluated their interaction in a normal cell milieu by immunoprecipitation. We transfected GFP-tagged full-length KSR1 into HEK293T cells and observed that KSR1 co-immunoprecipitated with YAP from HEK293T cells (Fig. 2D). YAP was not detected in samples precipitated from cells with IgG nor from cells transfected with GFP only. These data reveal that KSR1 and YAP interact in cells.

To verify the binding of KSR1 to YAP in another cell type, we carried out similar experiments in MEFs. MEFs express appreciable amounts of YAP, but little KSR1^32^. Therefore, binding studies were performed in MEFs that overexpress KSR1. The amount of KSR1 in KSR1^+/+^ MEFs is approximately 70-fold greater than that in WT MEFs (Supplementary Fig. 2A and B). KSR1 co-immunoprecipitated with endogenous YAP from KSR1^+/+^ MEFs (Fig. 2E). The absence of KSR1 from the negative IgG control demonstrates binding specificity. Collectively, our data indicate that KSR1 binds MST1, LATS1 and YAP, the kinases and transcriptional module of the Hippo pathway.

### KSR1 is required for maximal YAP co-transcriptional function

**Fig. 3:**
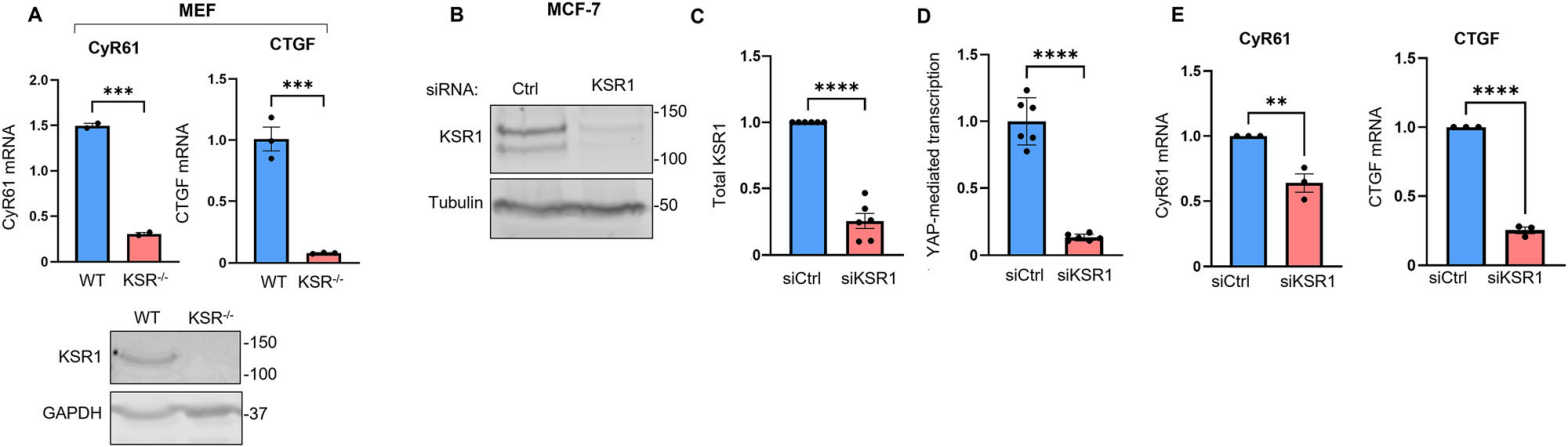
KSR1 regulates YAP co-transcriptional function. **A** Total RNA was extracted from WT and KSR1^–/–^ MEFs. CyR61 and CTGF hnRN were measured by quantitative RT-PCR and corrected for GAPDH hnRNA in the same sample. Data represent the means ± S.E. (error bars) of two to three independent experiments, each performed in triplicate. In parallel, equal amounts of protein lysate from WT and KSR1^−/−^ MEFs were resolved by Western blotting (lower panel) and probed with antibodies to KSR1 and GAPDH (loading control). **B** MCF-7 TEAD reporter cells were transfected with control (Ctrl) or KSR1-specific siRNA for 72 h. Cells were lysed, and equal amounts of protein were resolved by SDS-PAGE and Western blotting. Blots were probed with antibodies to KSR1 and tubulin (control). **C** The bands in panel B were quantified, and the amount of KSR1 was corrected for the loading control in the same sample. Data are expressed as means ± S.E. (error bars), with cells transfected with control siRNA set as 1 (*N* = 6). **D** Luciferase activity was quantified using the ONE-Step™ Luciferase Assay System. Data are means ± SD from two separate biological samples, each performed in three technical replicates (*N* = 2, *n* = 6). **E** Total RNA was extracted from MCF-7 cells transfected with control (siCtrl) or KSR1-specific siRNA (siKSR1). CyR61 and CTGF hnRNA were quantified as described for panel A. Data represent the means ± S.E. (error bars) of three independent experiments, each performed in triplicate. **p < 0.01, ***p < 0.001, ****p < 0.0001, Student’s *t* test.

YAP co-transcriptional activity is influenced by its binding partners^1^. To investigate whether binding to KSR1 modulates YAP function, we conducted RT-PCR to quantify the transcription of YAP target genes. MEFs lacking KSR1 were compared to control MEFs. Our data revealed that knocking out KSR1 substantially reduced the mRNA expression of two YAP target genes, CyR61 and CTGF, by 80% and 93%, respectively (Fig. 3A), indicating that YAP function is attenuated in the absence of KSR1.

YAP activity drives fundamental aspects of tumorigenesis in breast carcinoma^3^. To verify our findings in another cell type and to investigate whether KSR1 regulates YAP activity in breast epithelium, we used siRNA to knock down KSR1 in MCF-7 human breast carcinoma cells that constitutively express a TEAD-responsive luciferase reporter (Fig. 3B). siRNA against KSR1 reduced KSR1 protein by 75% (Fig. 3B, C). YAP co-transcriptional function was decreased by 87% when KSR1 was knocked down (Fig. 3D). Moreover, the transcription of YAP target genes CyR61 and CTGF was significantly reduced (Fig. 3E). Overall, our data indicate that KSR1is required for full YAP co-transcriptional function.

### KSR1 regulates YAP expression

**Fig. 4:**
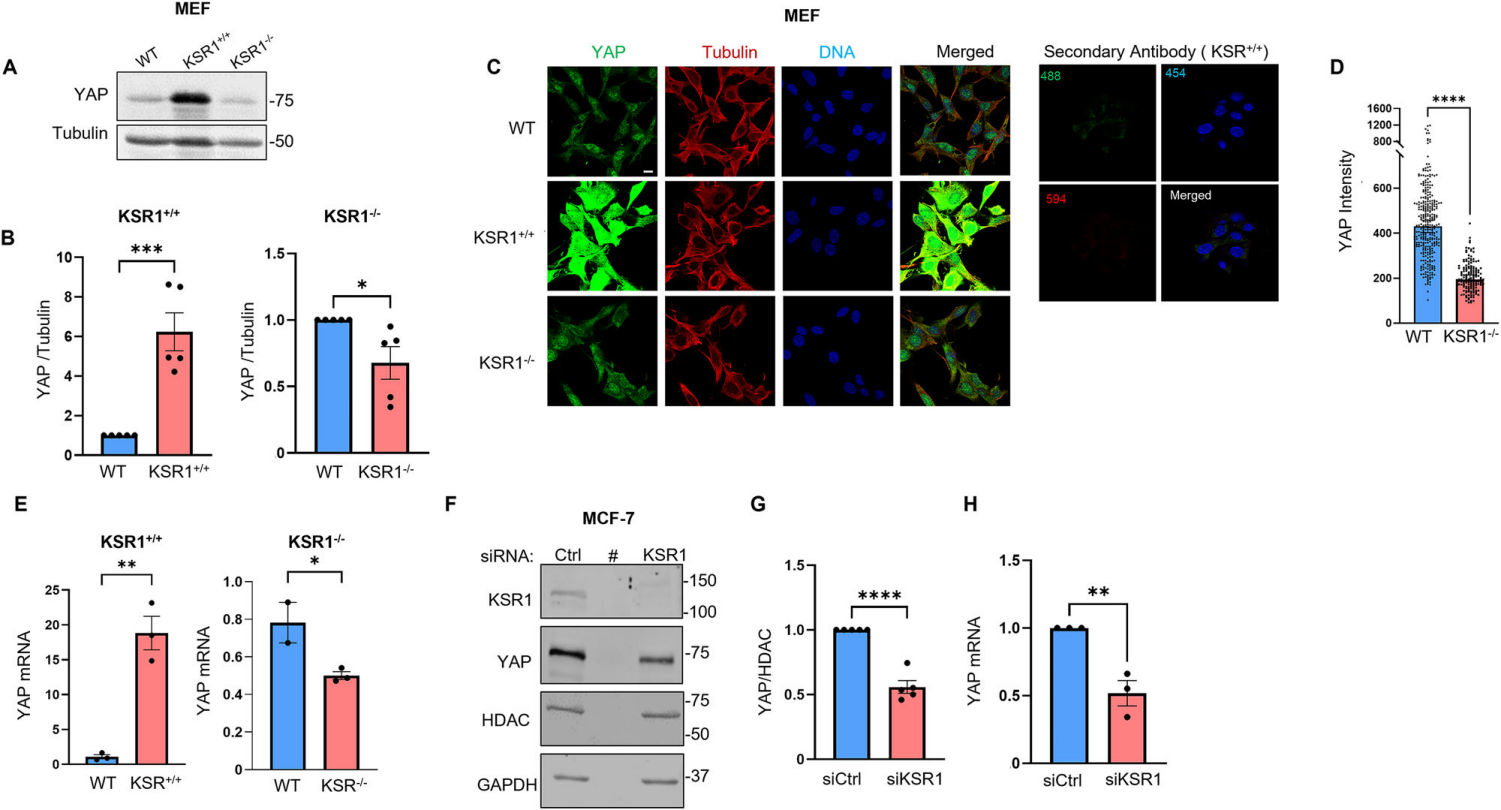
KSR1 regulates YAP expression. **A** Equal amounts of protein lysate from WT, KSR1^+/+^ and KSR1^−/−^ MEFs were resolved by Western blotting. Blots were probed with anti-YAP and anti-β-tubulin antibodies (loading control). Data represent ≥5 independent experiments. **B** The amount of YAP expression in panel A was quantified and corrected for β-tubulin in the corresponding sample. Data are expressed as means ± S.E. (error bars), with WT MEFs set as 1 (*N* = 5). **C** Cells were fixed and stained with anti-YAP monoclonal antibody (*green*) and anti-β-tubulin antibodies (*red*). DNA was stained with Hoechst (*blue*). The merged image displays an overlay of YAP, tubulin and DNA staining. KSR1^+/+^ MEFs incubated with only secondary antibodies are controls (right panels). Data are representative of ≥100 cells from three biological samples imaged with a Zeiss LSM880 confocal microscope using a 63× objective lens. Scale bar, 10 μm. **D** Tubulin staining was used to identify and separate cells. Each cell was segmented using Cellpose (Python v.3.10). The intensity of YAP was quantified. Data are means ± S.E. (*N* = 3, 150 cells). ****p < 0.0001, Mann-Whitney test. **E** Total RNA was extracted from WT, KSR1^+/+^ and KSR1^–/–^ MEFs. YAP hnRNA was measured by quantitative RT-PCR and corrected for GAPDH hnRNA in the same sample. Data represent the means S.E. (error bars) of two to three independent experiments, each performed in triplicate. **F** MCF-7 cells were transfected with control (Ctrl) or KSR1 specific siRNA. Cells were lysed, resolved by Western blotting and probed with antibodies to KSR1, YAP, HDAC and GAPDH (control lane). # indicates an empty lane (**G**) Bands in (**F**) were quantified and the amounts of YAP was corrected for HDAC in the same sample. Data are expressed as means ± S.E. (error bars), with cells transfected with control siRNA set as 1 (*N* = 5). **H** Total RNA was extracted from cells and YAP hnRNA was measured by quantitative RT-PCR and corrected for GAPDH hnRNA in the same sample. Data represent the means S.E. (error bars) of three independent experiments, each performed in triplicate. *p < 0.05, **p < 0.01, ***p < 0.001, ****p < 0.0001, Student’s *t* test.

To gain mechanistic insight  into how KSR1 regulates YAP function, we evaluated the amount of YAP protein in MEFs overexpressing or lacking KSR1. Western blotting revealed that the amount of YAP protein is 6 ± 0.9-fold greater in KSR1^+/+^ MEFs than in WT MEFs (Fig. 4A, B, *left panel*). We validated the increase in total YAP in KSR1^+/+^ MEFs using immunofluorescence staining with a specific anti-YAP antibody (Fig. 4C). By contrast, overexpression of KSR1 does not substantially alter the amounts of TEAD or RhoA protein (Supplementary Fig. 2C). Western blotting of lysates from KSR1^−/−^ MEFs showed that depleting KSR1 reduces total YAP by 30% (Fig. 4A, B, *right panel*). Confocal microscopic images confirmed the significant reduction in YAP when KSR1 is depleted from MEFs (Fig. 4C, D). Additionally, we assessed the post-translational modification of YAP by evaluating its phosphorylation at S^127^. Our data indicate that despite an 8.8-fold increase in total YAP levels, the pYAP/YAP ratio is only modestly greater in KSR1^+/+^ than in KSR1^−/−^ MEFs (Supplementary Fig. 2E–G). Taken together, these data revealed that KSR1 regulates YAP protein levels.

To determine whether KSR1 regulates YAP at the protein or mRNA level, we quantified mRNA by RT-PCR in WT, KSR1^+/+^ and KSR1^–/–^ MEFs. The amount of YAP mRNA in KSR1^+/+^ MEFs was 18.8 ± 2.4-fold greater than in control MEFs (Fig. 4E, *left panel*), while YAP mRNA is reduced by 37% in KSR1^−/−^ MEFs (Fig. 4E, *right panel*). Note that TAZ mRNA is slightly, albeit significantly, higher in KSR1^–/–^MEFs than WT MEFs (Supplementary Fig. 2D), likely as a compensatory response to the reduction in YAP mRNA.

To ascertain whether the reduced YAP protein and mRNA seen in KSR1^−/−^ MEFs occur in another cell line, we knocked down KSR1 in MCF-7 cells. Consistent with our observations in MEFs, knocking down KSR1 significantly reduced total YAP protein (Fig. 4F, G) and mRNA (Fig. 4H) by 45% and 51%, respectively. Overall, these data suggest that KSR1 selectively regulates YAP mRNA expression.

To determine whether KSR1 and YAP mRNA correlate, we analyzed data from biopsies reported in The Cancer Genome Atlas (TCGA, PanCancer Atlas). We observed a weak, but statistically significant, positive correlation between KSR1 and YAP mRNA from 10,967 patient samples across all cancer types (Supplementary Fig. 3), supporting a functional correlation between KSR1 and YAP in malignancy.

### KSR1 regulates YAP subcellular localization

**Fig. 5:**
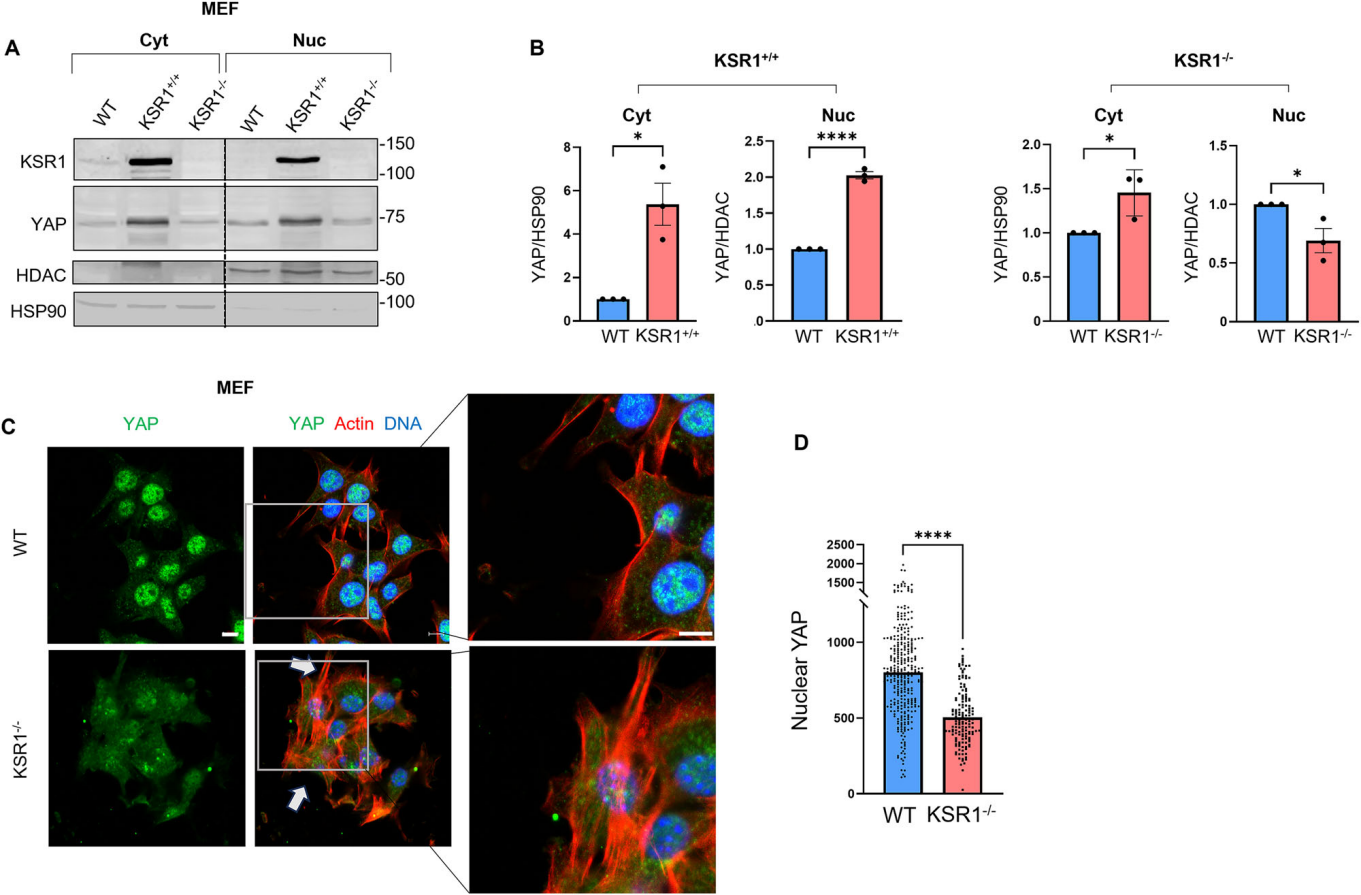
KSR1 regulates YAP subcellular localization. **A** WT, KSR1^+/+^ and KSR1^−/−^ MEFs were lysed and fractionated. Equal amounts of cytoplasmic (Cyt) and nuclear (Nuc) protein were resolved by Western blotting and probed with the indicated antibodies. HSP90 and HDAC were controls for cytoplasmic and nuclear fractions, respectively. **B** YAP bands were quantified and corrected for HSP90 (cytoplasmic fraction) or HDAC (nuclear fraction). Data are expressed as means ± S.E. (*N* = 3), with WT MEFs set to 1. *p < 0.05, ****p < 0.0001, Student’s *t* test. **C** WT and KSR1^–/–^ MEFs were fixed and stained with anti-YAP monoclonal antibody (*green*). Actin was visualized using Alexa Fluor® 568 Phalloidin (red) and DNA was stained with Hoechst 33342 (blue). Representative confocal images are shown from at least 100 cells imaged from three separate biological samples. Insets show higher magnification of the selected areas (right panels). Scale bar, 10 μm. White arrows show areas of actin protrusions and cell aggregates. **D** Python (v.3.10) was used to identify and segment the nuclear regions based on Hoechst staining. The intensity of YAP staining in the nucleus in each cell was quantified. Data are means ± S.E. (*N* = 3, 150 cells). ****p < 0.0001, Mann–Whitney test.

YAP subcellular localization is influenced by both the active state of Hippo kinases and by YAP binding partners^1^. Based on our observation of KSR1:YAP complex formation in cells, we explored whether KSR1 influences the subcellular localization of YAP. WT, KSR1^+/+^ and KSR1^–/–^ MEFs were separated into cytoplasmic and nuclear fractions (Fig. 5A). The amount of YAP in both cytoplasmic and nuclear fractions in KSR1^+/+^ MEFs is significantly higher than that in WT cells (Fig. 5A, B, *left panels*). KSR1^–/–^ MEFs have more YAP in the cytoplasm, but less in the nucleus, than WT cells (Fig. 5A, B, *right panels*). In a complementary approach, we quantified YAP nuclear intensity in confocal images from WT and KSR1^–/–^ cells (Fig. 5C). Quantifying confocal microscopic images confirmed a 38% reduction in the signal intensity of nuclear YAP in KSR1^–/–^ MEFs (Fig. 5D). This observation suggests that YAP is retained in the cytoplasm in the absence of KSR1. Overall, these data indicate that KSR1 positively regulates YAP expression and nuclear localization.

### KSR1 modulates YAP via RhoA and the actin network

To gain mechanistic insight into the pathways modulated by KSR1 and to identify the mediators underlying YAP regulation by KSR1, we conducted proteomic screening of cell lysates using reverse phase protein arrays (RPPA). RPPA is a multiplexed, antibody-based relative quantification of specific cellular proteins with their post-translational modifications^33^. We compared changes in protein expression and/or activation between WT, KSR1^+/+^ and KSR1^–/–^ MEFs (Supplementary Fig. 4). Principal component analysis (PCA) demonstrated distinct molecular profiles for WT and either KSR1^+/+^ or KSR1^−/−^ (Supplementary Fig. 4A, D). We selected the top 10 proteins that were significantly altered by at least two-fold in KSR1^+/+^ or

KSR1^–/–^ cells (Supplementary Fig. 4B, E). Our data reveal that altered levels of KSR1 protein results in molecular changes across multiple pathways. To evaluate the interaction network of KSR1 and its relevance to the Hippo core components, we used the Systematic Protein Association Dynamic ANalyzer (SPADAN)^34^, a novel algorithm that allows a holistic insight into the dynamics of protein interactions and provides quantitative predictions of system behavior. We extracted from the SIGNOR database a list of KSR1 interacting proteins, which overlapped with the list of the first 100 ranked proteins extracted from RPPA. We assessed the interaction types, such as activation or inhibitory effects. Consistent with our experimental observations, we identified, MST1 (*STK4*) as a main hub connecting KSR1 to YAP (Supplementary Fig. 5).

**Fig. 6:**
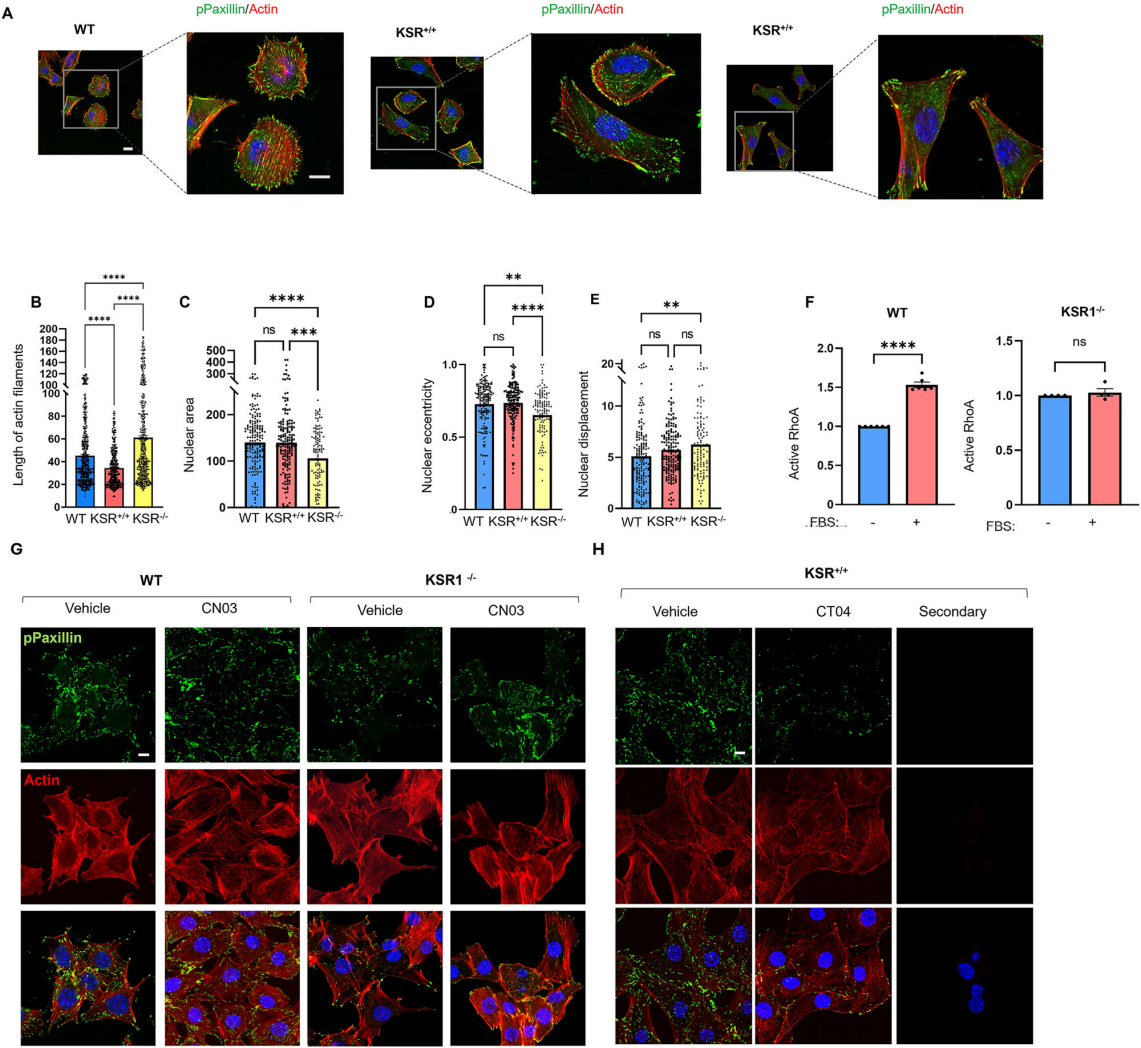
KSR1 modulates YAP function via RhoA and the actin network. **A** WT, KSR1^+/+^ and KSR1^−/−^MEFs were fixed and stained with anti-phosphoPaxillin^Tyr118^ (pPaxillin) monoclonal antibody (*green*) to visualize focal adhesions. Actin was visualized using Alexa Fluor® 568 Phalloidin (*red*), and DNA was stained with Hoechst (*blue*). Representative confocal images are shown from at least 100 cells imaged from three separate biological samples. Insets show higher magnification of the boxed area. Scale bar, 10 μm. **B** Actin stress fibers were segmented and measured as described in the methods. ****p < 0.0001, Kruskal-Wallis test. **C**–**E** Nuclear regions were segmented based on Hoechst staining and geometric parameters of nuclear segments, including area, eccentricity (ratio of minor axis length to major axis length) and solidity (ratio of the region area to area of the convex hull), were measured. The extent of nuclear displacement is defined as the Euclidean distance between the nuclear centroid and the cytoplasmic centroid. Data are expressed as medians ± 95% CI (**B**) and means ± S.E. **C**–**E** from at least 100 cells measured in three separate biological samples. **p < 0.01, ***p < 0.001, ****p < 0.0001, Kruskal-Wallis test. ns, not significant. **F** WT (*left panel*) and KSR1^−/−^ (*right panel*) MEFs starved of serum for 16 h were incubated with (+) or without (–) FBS for 5 min. RhoA-GTP (active RhoA) was quantified using the G-LISA assay. Data represent means ± S.E., ****p < 0.0001, Student’s *t* test (*N* ≥ 4 biological samples), with values in starved cells set to 1. **G** WT and KSR1^–/–^ MEFs were starved of serum for 6 h, then incubated with either vehicle (ddH2O) or the RhoA activator CN03 for 4 h. Cells were fixed and processed as described for (**A**). **H** Serum-starved KSR1^+/+^ MEFs were incubated with the RhoA inhibitor CT04 or vehicle (ddH_2_O) for 4 h. Cells were processed as described for (**A**). All confocal images are representative of at least 100 cells imaged from three separate biological samples. Scale bar, 10 μm.

Analysis of protein clusters revealed that proteins associated with DNA repair and cell adhesion are altered in KSR1^+/+^ and KSR1^–/–^ MEFs, respectively (Supplementary Fig. 4C, F). Altered cell adhesion in KSR1^−/−^ MEFs is congruent with our observations that KSR1^–/–^ MEFs exhibit altered cell morphology, generate elongated cell protrusions and tend to form clusters (Fig. 5C, arrows). To address whether these morphological changes are due to an altered actin network, we investigated potential alterations in the organization of actin and focal adhesion assembly. The organization of functional focal adhesions, evaluated with phosphorylation of paxillin on Tyr118^35^, in WT MEFs was similar to that in KSR1^+/+^ MEFs (Fig. 6A). In contrast, in KSR1^–/–^ MEFs, the typical dispersed distribution pattern of focal adhesions is altered, and focal adhesions are aggregated at the sites of actin projections. Actin filaments are significantly longer in KSR1^–/–^ MEFs and shorter in KSR1^+/+^ cells than in WT MEFs (Fig. 6A, B). We measured nuclear area and nuclear eccentricity, which are indices of nuclear shape. The nuclear areas of KSR1^+/+^ and WT MEFs are similar (Fig. 6C), but are significantly reduced in KSR1^–/–^ MEFs (Fig. 6C). In addition, nuclear eccentricity is significantly lower and nuclear displacement is significantly higher in KSR1^–/–^ MEFs than in WT cells (Fig. 6D, E), which are suggestive of altered mechanical cues in KSR1^–/–^ MEFs. Given the critical role of YAP in mechanotransduction and the impact of actin organization on YAP nuclear localization and function^36,37^, these observations suggest a possible role of KSR1 in modulating actin organization and nuclear architecture.

RhoA regulates the actin cytoskeleton, particularly in relation to cell shape changes and adhesion dynamics^38^. Moreover, RhoA increases YAP nuclear translocation and enhances the expression of YAP target genes^39^. To evaluate whether the observed changes in actin organization and YAP subcellular localization are influenced by RhoA, we assessed the effect of serum on the amount of active (GTP-bound) RhoA in cells with different amounts of KSR1. We observed that serum increases active RhoA in WT MEFs, but has no effect on KSR1^−/−^ MEFs (Fig. 6F). WT, KSR1^+/+^ and KSR1^−/−^ MEFs have similar amounts of total RhoA protein (Supplementary Fig. 2C). Therefore, KSR1 is necessary for maximal increase in active RhoA in response to serum stimulation.

To test the hypothesis that the impact of KSR1 on nuclear localization of YAP is mediated by RhoA, we incubated WT and KSR1^−/−^ MEFs with a RhoA activator. CN03 blocks GTP hydrolysis and increases active RhoA without altering active Rac or Cdc42^40,41^. We investigated the impact of CN03 on the actin cytoskeleton and focal adhesions in our experimental model. We confirmed that CN03 significantly increased the amount of active RhoA in WT and KSR1^−/−^ MEFs (Supplementary Fig. 6). While CN03 did not appreciably change focal adhesions in WT cells, in KSR1^−/−^ MEFs it increased the formation of focal adhesions, suppressed cell aggregates and enhanced areas of cell-cell contact (Fig. 6G). Moreover, inhibiting RhoA with CT04 in KSR1^+/+^ MEFs reduced the formation of focal adhesions (Fig. 6H). These data indicate that RhoA is a regulator of cell-substratum contact and modulates the formation of focal adhesions. Moreover, the results show that KSR1 regulates cell adhesion through the RhoA/actin network.

**Fig. 7:**
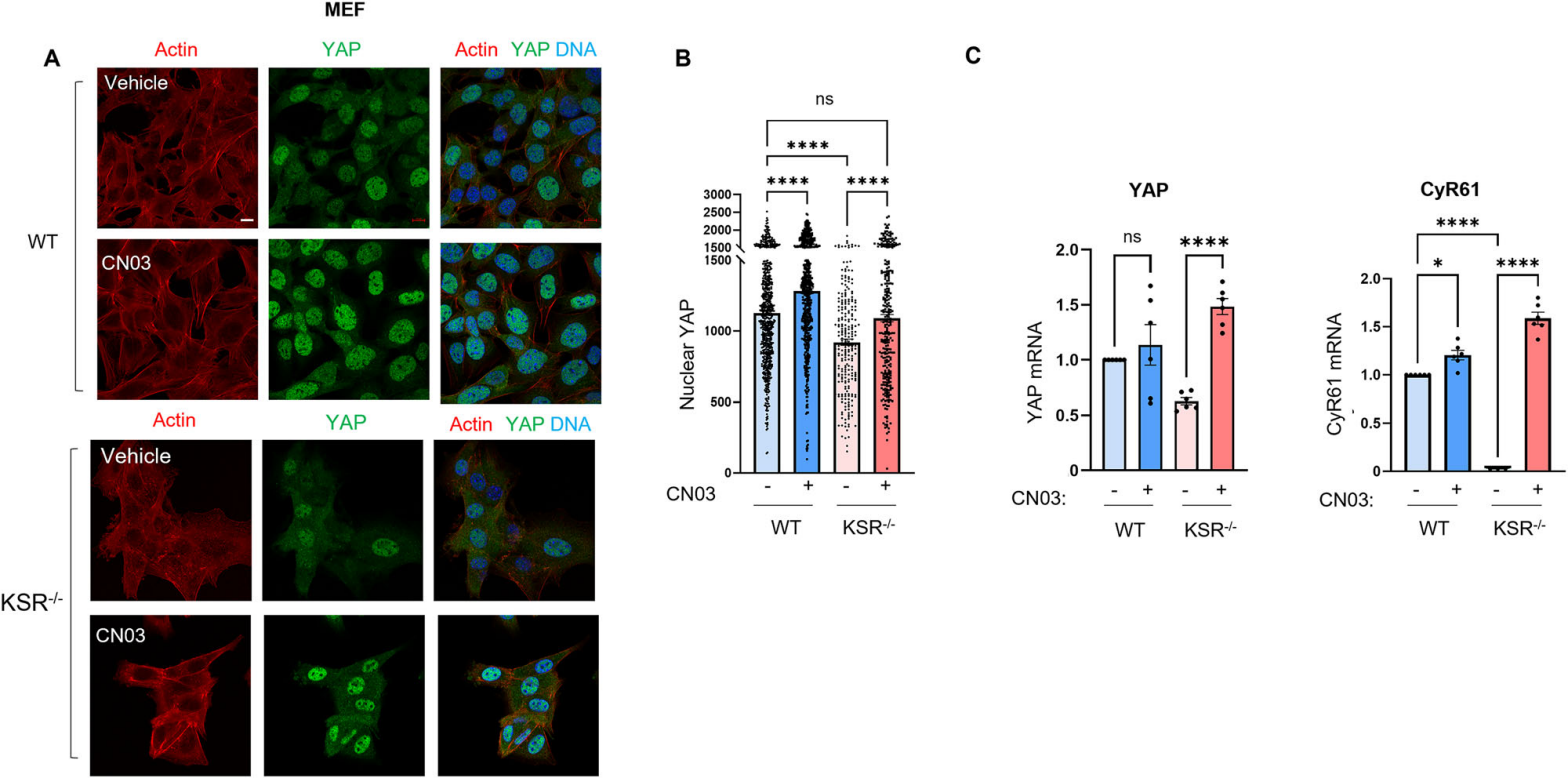
Active RhoA restores YAP co-transcriptional functions in KSR1^−/−^ MEFs. **A** WT and KSR1^−/−^ MEFs were starved of serum for 6 h, then incubated with vehicle (ddH_2_O) or the RhoA activator CN03 for 4 h. Cells were fixed and stained with anti-YAP monoclonal antibody (*green*). Actin was visualized using Alexa Fluor® 568 Phalloidin (*red*), and DNA was stained with Hoechst (*blue*). Representative confocal images are shown from at least 100 cells imaged from three separate biological samples. Scale bar, 10 μm. **B** Nuclear regions were segmented based on Hoechst staining and YAP intensity was measured in the nuclear segments. Data are expressed as means ± S.E. from at least 250 cells measured from three separate biological samples. ****p < 0.0001, Kruskal-Wallis test. ns not significant. **C** WT and KSR1^−/−^ MEFs were incubated with either vehicle (–) or the RhoA activator CN03 (+) for 16 h in serum-free medium. Total RNA was extracted, and quantitative RT-PCR was performed to measure YAP and CyR61 hnRNA. The amount of hnRNA was corrected for GAPDH hnRNA in the same sample. Data represent the means ± S.E. (error bars) of two independent experiments, each performed in triplicate. *p < 0.05; ****p < 0.0001, ANOVA; ns not significant. WT MEFs incubated with vehicle were set to 1.

Next, we assessed whether increasing the amount of active RhoA rescues the reduced YAP nuclear localization that we observed in KSR1^−/−^ MEFs. Using confocal microscopy, we quantified the amount of nuclear YAP in cells incubated with CN03. RhoA activation significantly increased the amount of YAP in the nuclei of both WT and KSR1^−/−^ MEFs (Fig. 7A, B). In KSR1^−/−^ MEFs, CN03 restored the amount of nuclear YAP (Fig. 7A, B) and significantly increased the mRNA levels of both YAP and CyR61 (Fig. 7C). Taken together, our data suggest that KSR1 regulates YAP in part through RhoA-mediated actin remodeling.

### KSR1 serves as an intersection node of crosstalk between Hippo and MAPK

**Fig. 8:**
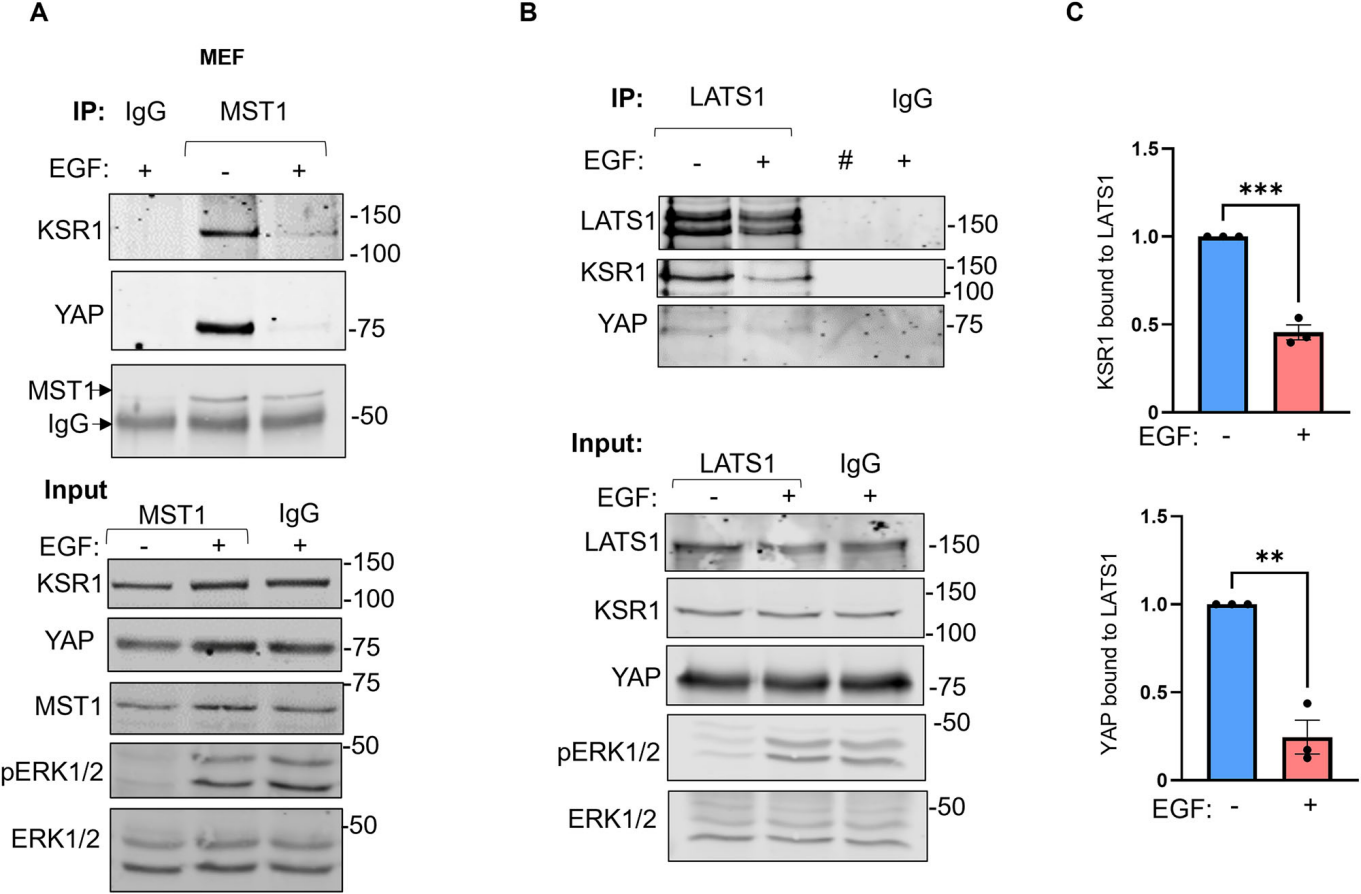
KSR1 serves as an intersection node of crosstalk between Hippo and MAPK. KSR1^+/+^ MEFs were serum-starved for 16 h, then incubated with 100 µg/mL EGF or vehicle (ddH2O) for 5 min. Cells were lysed and immunoprecipitated with anti-MST1 antibodies (**A**) or anti-LATS1 antibodies (**B**). Rabbit IgG was the negative control. Immune complexes were analyzed by Western blotting. Blots were probed with antibodies to KSR1, YAP, MST1, LATS, ERK1/2 and phosphorylated ERK1/2 (pERK1/2). All blots are representative of at least three independent experiments. # indicates an empty lane. **C** The IP bands in (**B**) were quantified, and the amount of KSR1 (upper panel) or YAP (lower panel) was corrected for the amount of LATS1 in the same sample. Data are expressed as means ± S.E. (error bars), **p < 0.01, ***p < 0.001, Student’s *t* test with values in no EGF set as 1 (*N* = 3).

KSR1 is a hub for the MAPK cascade, coordinating signaling events^13,42^. Our data presented here identify KSR1 as a scaffold of the Hippo pathway and raise the question of whether KSR1 facilitates crosstalk between the Hippo and MAPK pathways. To explore this possibility, we investigated whether EGF, which activates the MAPK cascade^11^, influences the association of KSR1 with components of the Hippo pathway. Incubating cells with EGF markedly impairs the ability of KSR1 to co-immunoprecipitate with MST1 (Fig. 8A). EGF also reduced the interaction between YAP and MST1 (Fig. 8A).

We assessed whether the KSR1 association with LATS1 is also regulated by EGF. EGF significantly reduced the amounts of both YAP and KSR1 bound to LATS1 by 76% and 55%, respectively (Fig. 8B, C). Probing Western blots of cell lysates for phosphorylated ERK verified that EGF activated the MAPK pathway (Fig. 8). Note that EGF did not alter the amount of MST1 or LATS1 that was immunoprecipitated (Fig. 8). Overall, these data demonstrate that EGF signaling regulates the ability of KSR1 to form a complex with MST1, LATS1 and YAP in cells, suggesting a crosstalk between the Hippo and MAPK pathways.

## Discussion

**Fig. 9:**
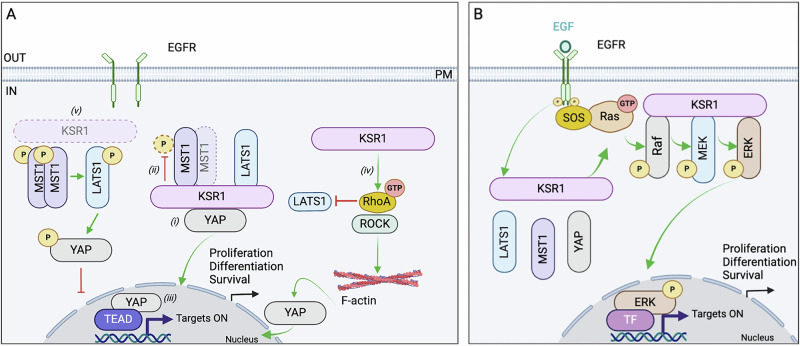
Model of KSR1 in Hippo signaling. **A** KSR1 constitutively binds MST1, YAP and LATS1, components of the Hippo pathway. (i) KSR1 binds directly to YAP, potentially protecting YAP from phosphorylation by kinases of the Hippo cascade, thereby enabling YAP to translocate to the nucleus. (ii) KSR1 binds directly to MST1 and interferes with MST1 dimerization and consequent autophosphorylation/activation. (iii) KSR1 enhances YAP mRNA expression. (iv) KSR1 enhances activation of RhoA and RhoA/Rho-associated protein kinase (ROCK). This modulates actin organization, suppresses LATS1 activation and promotes YAP nuclear localization. (v) Depletion of KSR1 increases the amounts of phosphorylated (active) MST1 and LATS1, which increases YAP phosphorylation and reduces YAP function by blocking its translocation to the nucleus. **B** In response to EGF, KSR1 scaffolds the MAPK pathway by assembling a functional complex of Raf, MEK and ERK. EGF attenuates the association of KSR1 with MST1, LATS1 and YAP, thus releasing the inhibitory impact of KSR1 on Hippo signaling. The KSR1 is then free to scaffold components of the MAPK cascade. Green arrows and red lines represent activation and inhibition, respectively. EGF epidermal growth factor, EGFR epidermal growth factor receptor, ROCK Rho-associated protein kinase, PM plasma membrane, TF transcription factor. The figure was generated in BioRender.

Scaffold proteins are molecular hubs for signal transduction. KSR1 is a recognized scaffold in the MAPK cascade^13,42^. In this study, we demonstrated that KSR1 is also a scaffold of the Hippo pathway, assembling a complex containing MST1, LATS1, and YAP. Using several diverse experimental approaches, we identified five potential mechanisms (Fig. 9A) through which KSR1 may suppress Hippo signaling and enhance YAP co-transcriptional activity:

(i) Direct binding of KSR1 to YAP could reduce YAP phosphorylation by the Hippo kinases. We mapped the site where KSR1 binds to the N-terminal region of YAP, which contains the WW and TEAD-binding domains. LATS1 was previously shown to bind to the WW domain of YAP and phosphorylate YAP^43^. It is feasible that when KSR1 binds to YAP it induces a steric hindrance or a conformational change that impairs the ability of LATS1 to bind the WW domain and catalyze YAP phosphorylation.
(ii) KSR1 may influence the activation of MST1. MST1 activation requires phosphorylation on Thr^183^ in the activation loop to initiate Hippo signaling^28^. The αG-helix is essential for the proper positioning of the MST1 catalytic residue Thr^183^. Several single-point mutations in the αG-helix have been reported to prevent MST1 dimerization and block Thr^183^ phosphorylation^26^, confirming that the αG-helix is located at the dimer interface and that MST1 undergoes trans-autophosphorylation, i.e., each monomer catalyzes phosphorylation of its partner^30^. We showed that MST1 phosphorylation is enhanced in the absence of KSR1, implying that KSR1 may inhibit MST1 phosphorylation. In support of this observation, our computational modeling suggests that KSR1 binding to the MST1 dimer interferes with optimal formation of the MST1:MST1 interface that is required for the activation loop of one monomer to enter the catalytic site of its partner. This could disrupt trans-phosphorylation and inhibit MST1 activation. Although our data show that KSR1 binds directly to MST1 and potentially influences MST1 activation, we cannot exclude the possibility that KSR1 affects MST1 activation through an indirect mechanism that is independent of direct binding.
(iii) IQGAP1, a scaffold protein of the Hippo pathway, binds directly to YAP^23^, the kinase module of the Hippo pathway^24^ and KSR1^17^. The complex of IQGAP1 and KSR1 modulates MAPK signaling by transphosphorylation, which is effected by the scaffold:scaffold interaction^17^. KSR1 may similarly influence Hippo signaling by binding IQGAP1 and the kinase module of the Hippo signaling pathway to regulate cellular responses.
(iv) We demonstrated that YAP mRNA levels are reduced in KSR1-null cells and increased in cells overexpressing KSR1, revealing that KSR1 regulates *YAP* gene transcription. Consistent with these observations, we identified a positive correlation between the mRNA levels of KSR1 and YAP across all cancer types. There are several possible mechanisms by which KSR1 may influence YAP mRNA expression. YAP regulates its own expression through a positive feed-forward mechanism, which involves activation of Myc and AP-1 transcription factors^44,45^. The reduced YAP mRNA expression in KSR1-deficient cells may be due to reduced abundance, cyoplasmic retention and/or decreased nuclear function of YAP, which may be influenced by KSR1-mediated MAPK signaling. KSR1 forms a functional protein complex with Merlin or AMOTL1/2^46,47^, which are regulators of YAP transcriptional activity. In particular, AMOTL2 regulates the availability of the YAP promoter and influences transcription of the YAP gene. Therefore, KSR1 may facilitate YAP gene transcription through its interaction with Merlin or AMOTL2. A second possibility is that KSR1 may directly influence transcription of the YAP gene^48^. While, KSR1 is predominantly localized in the cytoplasm, nuclear shuttling has been reported under certain conditions, such as phosphorylation of KSR1^49^. Our data reveal that MEFs overexpressing KSR1 have substantial accumulation of KSR1 in the nuclear fraction (Fig. 5A). Published studies show that KSR1 regulates mRNA expression of several proteins, including epithelial stromal interaction 1 and N-cadherin^50^. Thus, nuclear KSR1 may directly or indirectly influence YAP gene transcription. Regardless of the mechanism, the findings presented here indicate that KSR1 modulates the amount of YAP mRNA.
(v) KSR1 could alter Hippo signaling via its effect on the actin cytoskeleton. The role of RhoA in enhancing YAP nuclear function is well-established^51^. Moreover, RhoA regulates expression of YAP mRNA in hepatocellular carcinoma cells^52^. We showed that cells lacking KSR1 have altered actin organization and decreased active RhoA, with a concomitant reduction in nuclear YAP abundance and activity. Our data indicate that increasing active RhoA partially compensates for the reduction in nuclear YAP that results from loss of KSR1. Importantly, guanine nucleotide exchange factor (GEF) H1, which modulates localized RhoA activation^53^, binds directly to KSR1^54^. Therefore, it is possible that KSR1 participates in the regulation of RhoA activation by forming a complex with GEFH1/RhoA.

We do not propose that KSR1 is the exclusive mediator of MST1:LATS1 or LATS1:YAP interactions. Cell-type- and context-dependent mechanisms may influence the role of KSR1 in regulation of the Hippo pathway as alternative scaffolds or parallel signaling axes may compensate for or modify KSR1 function as a scaffold of Hippo signaling.

YAP regulates focal adhesions and actin orientation^55^, essential components of mechanotransduction^56,57^. Previous reports have shown that shRNA knockdown of KSR1 substantially reduces focal adhesion assembly in cells^46,58^. Consistent with these findings, we demonstrated that KSR1 regulates the formation of focal adhesions and nuclear morphology, which are indicative of regulation of mechanosensation^59,60^. Our RPPA analysis supports our experimental observations, identifying the cell adhesion network as one of the major pathways regulated by KSR1. Thus, our data uncover a role for KSR1 in regulating mechanotransduction upstream of RhoA, actin and YAP.

The interplay between EGF and Hippo signaling is poorly understood^61^. The published effects of EGF on YAP vary. Some studies report that when Hippo signaling is ON, EGF stimulation neither reduces levels of phosphorylated YAP nor enhances nuclear YAP localization^31,62^. In contrast, Fan et al. demonstrated that EGF inhibits Hippo signaling, thereby promoting YAP nuclear translocation and co-transcriptional activity^63^. Furthermore, the EGF receptor induces tyrosine phosphorylation of MOB1 and inhibits LATS1/2, in an MST1-independent manner, which in turn augments YAP co-transcriptional activity^61^. The reason for these disparate observations is unknown. Here, we identified a previously unrecognized potential regulatory mechanism linking MAPK and Hippo whereby EGF reduces the interaction between KSR1 and components of the Hippo pathway (see “model” Fig. 9B). We demonstrated that KSR1 is in a complex with MST1 and YAP in starved cells, i.e., when Hippo is ON (Fig. 9B). This constitutive binding of KSR1 to components of the Hippo cascade is regulated by EGF. By contrast to the role of EGF in assembling MAPK components, EGF stimulation of cells decreases the complex of KSR1, MST1, LATS1 and YAP. This association releases the inhibitory effect of KSR1 on Hippo signaling. The KSR1 is then free to potentially scaffold MAPK (Fig. 9B). Our findings suggest a mechanism through which EGF may influence the role of KSR1 as a scaffold of the Hippo signaling pathway. Collectively, our results identify KSR1 as a potential therapeutic target for modulating Hippo signaling.

## Materials and methods

### Materials

All reagents for tissue culture were from Life Technologies. Protein A-Sepharose was purchased from GE Healthcare. PVDF membranes were from Millipore Corp. RhoA activator II (#CN03) and RhoA inhibitor I (CT04) were purchased from Cytoskeleton. Blocking buffer (927-80001) and IR dye-conjugated (IRDye) secondary antibodies (anti-rabbit 926-32213, and anti-mouse 926-68022) were purchased from LI-COR Biosciences. Antibodies and dilutions are listed in Table 1.

**Table1:** Antibodies used in this study

Protein detected	Source, Catalog number, Clone number, Lot number	Dilution
Actin	Life technologies, # A12380, #1755955	IF:1/250
GAPDH	Cell Signaling Technology, #5174, D16H1, #8	WB: 1/1000
HDAC2	Cell Signaling Technology, #5113, 3F3, #4	WB: 1/1000
HSP90	Cell Signaling Technology #4877, C45G5, #5	WB: 1/1000
KSR1	Abcam, # (ab68483), EPR2421Y, #1001675-13	WB:1/1000
LATS1	Cell Signaling Technology, #3477, C66B5, #9	IP: 1/50 WB:1/1000
LATS1	Invitrogen, #711149, 2HCLC, #SB246686	IF:1/200
p44/42 MAPK (ERK1/2)	Cell Signaling Technology, #4696, L34F12, #29	WB:1/1000
Phospho-p44/42 MAPK (ERK1/2)^(T202/Y204)^	Cell Signaling Technology, #4376, 197G2, #17	WB:1/1000
MST1	Cell Signaling Technology, # 3682, #2	IP: 1/50 WB:1/1000
phosphoMST1^T183^	Cell Signaling Technology, #49332, E7U1D, #1	WB:1/1000
phosphoPaxillin^Y118^	Cell Signaling Technology, #69363, E9U9F, #1	IF: 1/100
RhoA	Cell Signaling Technology, #2117, 67B9, #6	WB:1/1000
panTEAD	Cell Signaling Technology, #13295, D3F7L, #1	WB:1/1000
β-tubulin	Millipore, # T5201, TUB 2.1.	WB: 1/1000 IF:1/100
YAP	Cell Signaling Technology, #12395, 1A12, #3	IF: 1/100
YAP	Cell Signaling Technology, #4912, #7	IP: 1/50
phosphoYAP^S127^	Cell Signaling Technology, #4911, #5	WB:1/1000

*WB* Western blotting, *IF* immunofluorescence, *IP* immunoprecipitation.

### Cell culture and transfection

HEK293T and MCF-7 cell lines were obtained from American Type Culture Collection. The MCF-7 TEAD reporter cell line was purchased from BPS biosciences (#60618). Mouse embryonic fibroblast (MEF) cells were isolated from embryonic day 14 embryos^64^. KSR1^+/+^ and KSR1^−/−^ cells, were kindly provided by Robert Lewis, University of Nebraska Medical Center^65^. MEFs and HEK-293 cells were grown at 37 °C with 5% CO_2_ in Dulbecco’s Modified Eagle Medium (DMEM), supplemented with 10% FBS. Cells were plated in 100-mm (for immunoprecipitation) or 60-mm (for fractionation) dishes. For experiments with the RhoA activator II (CN03) or RhoA inhibitor (CT04), cells were cultured for 48 h before they were starved of serum for 16 h and incubated with RhoA activator II (CN03) or RhoA inhibitor (CT04) for 6 h. All experiments were performed at ∼80% confluence. HEK293T cells were transfected using Lipofectamine 2000 (Invitrogen) according to the manufacturers’ instructions.

### Expression and purification of proteins

PCR product of MST1 was generated using Flag-tagged MST1 (Addgene, #1965) as template, then digested with BamHI and XhoI and subsequently inserted into the pGEX4T-TEV vector at the BamHI and XhoI sites to generate GST-MST1. GST-MST1 and GST-YAP (full-length, N-, M- and C-fragments) were expressed in *E. coli* and isolated using glutathione-Sepharose, essentially as previously described^23^. The purity and integrity of all purified proteins were validated by Coomassie blue-stained gels. KSR1 was synthesized with the T_N_T Quick Coupled Transcription/Translation system (Promega). The kit was used according to the manufacturer’s instructions, using 1 μg of plasmid as template.

### Pull-downs with GST-tagged constructs

For pull-downs of T_N_T products, samples were incubated with glutathione-Sepharose beads coated with the indicated protein for 3 h at 4 °C in 1 ml Buffer A (150 mM NaCl, 50 mM Tris-HCl, pH 7.4, and 1% Triton X-100). Beads were washed five times with Buffer A. Proteins bound to the beads were eluted by resuspending them in Laemmli sample buffer and incubating at 95 °C for 5 min. Samples were separated by SDS-PAGE and analyzed by Western blotting. For pull-downs from cell lysates, cells were washed with ice-cold PBS and lysed by sonication in Buffer A supplemented with protease and phosphatase inhibitors. Insoluble fractions were pelleted at 15,000 *g* for 10 min at 4 °C. Equal amounts of protein from cell lysates were pre-cleared and subjected to pull-down as described above.

### Immunoprecipitations

Cells were plated in 10-cm dishes to reach 80% confluence. The following day, the cells were washed with ice-cold PBS and lysed with 500 μl of Buffer A. Lysates were subjected to two rounds of sonication for 10 s each, and insoluble material was precipitated by centrifugation at 20,000 × *g* for 10 min at 4 °C. Supernatants were precleared with glutathione-Sepharose beads for 1 h. 5% of the supernatant was used as input. Equal amounts of protein lysate were incubated with protein A-Sepharose beads and anti-YAP or anti-MST1 polyclonal or anti-LATS1 monoclonal antibodies for 3 h at 4 °C. Rabbit IgG was used as controls for immunoprecipitation. Samples were washed five times with Buffer A, resolved by SDS-PAGE, and transferred to Polyvinylidene Fluoride (PVDF). Blots were probed with the indicated primary antibodies, followed by the appropriate IRDye-conjugated secondary antibodies or Quick Western (LI-COR, #926-69100), then scanned using the Odyssey imaging system.

### Immunostaining

Control, KSR1^+/+^ and KSR1^−/−^ MEFs were plated on ibidi µ slides (Ibidi, # 80826) and incubated at 37 °C with 5% CO_2_ in DMEM, supplemented with 10% FBS. For experiments with RhoA activator II (CN03) or RhoA inhibitor (CT04), cells were cultured for 48 h before they were starved of serum for 16 h and incubated with CN03 or CT04 for 6 h. The slides were washed with ice-cold PBS, then fixed in 4% paraformaldehyde for 15 min at 22 °C. Cells were permeabilized in 0.25% (v/v) Triton-X 100 for 10 min and blocked for 1 h in 1% (w/v) bovine serum albumin at 22 °C. Cells were incubated with the antibodies indicated in the figure legends. Cells incubated with secondary antibodies only were used as control. Unbound antibody was removed by washing five times with PBS, followed by incubation for 2 h with Alexa Fluor 488-labeled anti-rabbit antibody (green; Invitrogen, #A11036) and Alexa Fluor 568-labeled anti-mouse antibody (red; Invitrogen, #A11029). Alexa Fluor 568 Phalloidin (Molecular Probes, #A12380) was used to visualize actin. After five washes with PBS, the cells were mounted with ProLong Glass Antifade mounting medium (Invitrogen, #P36980). Cells were examined with a Zeiss LSM 780 confocal microscope using a 63× objective lens.

For nuclear, cytoplasmic, and actin quantification, nuclear regions were segmented from median filter denoised Hoechst images based on an Otsu’s auto-threshold (scikit-image, Python v3.10)^66^. Cytoplasmic regions were segmented based on actin or tubulin or actin fluorescence intensity using pre-trained Cellpose deep-learning segmentation models^67^. Cellpose is a generalist algorithm for cellular segmentation^68^. Actin stress fibers were segmented using a trained Random-Forest pixel classifier (scikit-learn; Python v 3.10), followed by a trained Random-Forest object classifier. Geometric parameters of the nuclear and cytoplasmic segments were collected, including area, eccentricity (ratio of minor axis length/major axis length), and solidity (ratio of the region area/area of the convex hull). The ratio of the nuclear to cytoplasmic area was calculated. The extent of nuclear displacement, defined as the Euclidean distance between the nuclear centroid and the cytoplasmic centroid, was calculated. Geometric parameters of the actin segments were collected, including area and major axis length. The percent area of actin stress fiber was calculated as actin area/cell area. At least 100 cells were examined for each condition.

For nuclear and cytoplasmic quantification, nuclear and cytoplasmic regions were segmented using pre-trained Cellpose deep-learning segmentation models^74^ (*nuclei* and *cyto2* models, respectively) and quantified using Python (v.3.10). Nuclear segments were based on Hoechst 33342 fluorescence intensity and cytoplasmic segments were based on a combination of YAP and phalloidin staining. Geometric parameters of the nuclear and cytoplasmic segments were collected, including area, eccentricity (ratio of minor axis length/major axis length), and solidity (ratio of the region area/area of the convex hull). The ratio of the nuclear to cytoplasmic area was calculated. The extent of nuclear displacement, defined as the Euclidean distance between the nuclear centroid and the cytoplasmic centroid, was calculated. Geometric parameters of the actin segments were collected, including area and major axis length.

Actin stress fibers were segmented using a trained Random-Forest pixel classifier (*scikit-learn*; Python v 3.10), followed by a trained Random-Forest object classifier. Geometric parameters of the actin segments were collected, including area and major axis length. The percent area of actin stress fiber was calculated as actin area/cell area. At least 100 cells were examined for each condition.

### Subcellular fractionation

Subcellular fractionation was conducted using the NE-PER^TM^ nuclear and cytoplasmic extraction kit (ThermoFisher, #78833), essentially as previously described^69^. Briefly, the cells pellets were resuspended in cytoplasmic extraction buffer and incubated on ice for 10 min. The lysates were then subjected to centrifugation at 16,000 × *g* for 5 min at 4 °C and the supernatant was collected as the cytoplasmic fraction. The cell pellets were resuspended in nuclear extraction buffer, then incubated on ice for 40 min. Samples were spun at 16,000 × *g* for 10 min at 4 °C, and the supernatant was collected as the nuclear fraction. The fractions were resolved by SDS-PAGE and Western blotting. Blots were probed with anti-YAP, anti-KSR1, anti-HDAC2, and anti-HSP90 primary antibodies, followed by the appropriate IRDye conjugated secondary antibodies, then scanned using the Odyssey imaging system.

### siRNA knockdown of KSR1 in MCF-7 cells

Two (A and B) human siRNA to KSR1 (#4392422, A: Assay ID: s16886 and B: Assay ID s16887) were purchased from Thermo Fisher Scientific and Silencer™ Negative Control siRNA (#AM4635), respectively. For optimal results, cells were transfected with siRNA A and B mixed (1:1) using RNAiMAX Lipofectamine reagent (Invitrogen), according to the manufacturer’s protocol.

The TEAD Luciferase Reporter MCF7 cell line (BPS-Bioscience) that stably expresses the firefly luciferase gene under the control of TEAD responsive elements was transfected with siControl or siKSR1 RNAs. 72 h after transfection, KSR1 knockdown was verified by Western blotting, and luciferase activity was detected using the ONE-Step™ Luciferase Assay System (BPS-Bioscience, #60690).

### Quantitative RT-PCR

To quantify YAP, TAZ, CTGF, and CyR61 hnRNA, cells were cultured for 24 h. Then total RNA was isolated from the cells using an RNA isolation kit (QIAGEN). 1 μg of RNA was reverse transcribed to cDNA using a High-Capacity cDNA Reverse Transcriptase kit (Applied Biosystems), according to the manufacturer’s instructions. RT-PCR was performed on a StepOnePlus Real Time PCR system (Applied Biosystems) using a TaqMan™ Gene Expression Assay (Thermo Fisher, #4331182) for YAP (ID# mouse:Mm01143263_m1; human: Hs00902712_g1), TAZ (ID#Mm01289583_m1), CyR61(ID# mouse:Mm00487498_m1; human: Hs00155479_m1) and CTGF (ID# mouse: Mm01192933_g1; human: Hs00170014_m1). RT-PCR enzyme activation was initiated for 10 min at 95 °C, then amplified for 40 cycles (15 s at 95 °C and 1 min at 60 °C). All samples were assayed in triplicate, and GAPDH (Thermo Fisher, mice, # 4352339E; human, #4326317E) was used as an internal control. Results were analyzed using the ΔΔCT method with StepOnePlus software (Applied Biosystems).

### RhoA G-LISA activation assay

Cells were cultured in six-well plates to reach 70% confluence and serum-starved for 16 h, then incubated with serum-containing medium for 5 min. Cells were lysed in the buffer provided in the RhoA G-LISA kit (Cytoskeleton, # BK121). Protein concentrations were measured with the Precision Red™ Advanced protein assay reagent (Cat. # ADV02) according to the manufacturer’s instructions. 30 μg of protein lysates were subjected to the G-LISA assay following the manufacturer’s protocol.

### Reverse-phase protein array

Control, KSR1^+/+^ and KSR1^−/−^ cells were cultured in DMEM, supplemented with 10% FBS. Three separate biological samples were collected and flash frozen in liquid N2. Cell lysates were analyzed at the RPPA core facility of the MD Anderson Cancer Center (Houston, TX, USA). Expression profiling data were analyzed using RStudio Cloud (https://www.rstudio.com/products/cloud/) version 4.1.2 (RStudio Team, 2022) software to find proteins differentially expressed between samples. Differential expression was represented by a protein-level fold change of minimum ±2 and *p* < 0.05 compared to control MEFs. STRING plots (https://string-db.org/) were generated using the first 100 highest-ranked differentially expressed genes showing ±2-fold expression changes, setting the organism to *Homo sapiens*.

### PPI networks (SPADAN) and computational modeling

We used the MATLAB toolbox for modeling protein-protein interaction (PPI) networks (SPADAN), incorporating experimentally validated PPI in SIGNOR 3.0 database. SPADAN was used to identify potential routes connecting KSR1 to YAP. These routes were subsequently scored to assess their alignment with the top 100 genes ranked by RPPA analysis. The score of each route includes: The scoring of each pathway was based on the following criteria: (1) the number of nodes within the pathway that correspond to the top 100 genes from the RPPA analysis, (2) the number of genes directly linked by a PPI edge to at least one of the pathway nodes and (3) the number of proteins from RPPA analysis of KSR1^+/+^ and KSR1^−/−^ MEFs, accessible via a single mediator, originating from at least one of the pathway nodes.

### Mass photometry

Mass photometry measurements were performed using a Refeyn OneMP (Refeyn Ltd, Oxford, UK). Recombinant human KSR1 (Abcam, #ab268719, lot #1064245‑2) and LATS1 (Active Motif, #81209, lot #32718001) proteins were diluted in PBS to a final concentration of 20 nM. Prior to measurement, the instrument optics were equilibrated at room temperature for at least one hour to ensure thermal stability of the optical path. Sample chambers were prepared on high-quality clean glass coverslips, and only buffer‑filtered protein solutions were used to minimize background scattering artifacts. Measurements were acquired in DiscoverMP software (Refeyn Ltd, Oxford, UK), recording mass landing events at single-molecule precision. Contrast signals were converted to molecular mass using instrument-specific calibration curves obtained with standard protein calibrants. Discrete peaks in the molecular mass histogram were fitted with Gaussian functions to determine peak centers and relative abundances of species present, allowing identification of monomeric versus oligomeric or complexed forms. Data were processed with DiscoverMP.

### Computational modeling

Models of the human MST1_2_ and MST1_2_:KSR1 complexes were obtained with AlphaFold2 multimer as implemented in the NIH HPC cluster (http://hpc.nih.gov). The main features of the complexes were well reproduced in all the predictions, and the top-ranked models were used for analysis. All the segments with a confidence score predicted local distance difference test (pLDDT) less than 60 were removed unless they contained fewer than 12 contiguous residues, in which case the segment was considered a loop, possibly too flexible or disordered to adopt a single conformation. The pLDDT scores, PAE matrices and xz coordinates of the complete structures are available in the Supplementary Fig. 1.

### In silico analysis of correlation between KSR1 and YAP mRNA expression in invasive breast carcinoma

mRNA expression z-scores (RNA Seq V2 RSEM) for KSR1 and YAP were generated using data from the Pan-Cancer Atlas available on cBioPortal.org, created by the TCGA Research Network (http://cancergenome.nih.gov).

### Statistics and reproducibility

Statistical analysis was performed by two-tailed Student’s *t* test, Mann-Whitney, Kruskal-Wallis or one-way analysis of variance (ANOVA) with Tukey’s post-hoc test as described in the figure legends, using Prism 9.5.1 (GraphPad). For all statistical analyses of Western blots, data represent three independent biological replicates. The number of technical replicates is provided in the corresponding figure legends.

### Miscellaneous methods

Western blotting images were quantified with Image Studio 2.0 (LI-COR Biosciences). Protein concentrations were determined using the Bio-Rad Protein Assay dye reagent (#500-0006). Quick Western Kit (LI-COR Biosciencesc#926-69100) was used to develop Western blots for MST1 immunoprecipitation studies.

### Reporting summary

Further information on research design is available in the Nature Portfolio Reporting Summary linked to this article.

## Supplementary information

Supplementary information

Description of Additional Supplementary Materials

Supplementary Data 1

Reporting Summary

## Data Availability

Original full blots and the numerical source data for graphs and charts have been submitted as supplementary information (Supplementary Data 1).

## References

[1] Ma, S., Meng, Z., Chen, R. & Guan, K. L. The Hippo pathway: biology and pathophysiology. *Annu. Rev. Biochem.* **88**, 577–604 (2019). 30566373 10.1146/annurev-biochem-013118-111829

[2] Fu, V., Plouffe, S. W. & Guan, K. L. The Hippo pathway in organ development, homeostasis, and regeneration. *Curr. Opin. Cell Biol.* **49**, 99–107 (2017). 29316535 10.1016/j.ceb.2017.12.012 PMC6348871

[3] Calses, P. C., Crawford, J. J., Lill, J. R. & Dey, A. Hippo pathway in cancer: aberrant regulation and therapeutic opportunities. *Trends Cancer* **5**, 297–307 (2019). 31174842 10.1016/j.trecan.2019.04.001

[4] Lo Sardo, F., Strano, S. & Blandino, G. YAP and TAZ in lung cancer: oncogenic role and clinical targeting. *Cancers* **10**,137 (2018). 10.3390/cancers10050137 PMC5977110 29734788

[5] Mouillet-Richard, S. & Laurent-Puig, P. YAP/TAZ Signalling in colorectal cancer: lessons from consensus molecular subtypes. *Cancers* **12**, 3160 (2020). 10.3390/cancers12113160 PMC7692643 33126419

[6] Silvis, M. R. et al. alpha-catenin is a tumor suppressor that controls cell accumulation by regulating the localization and activity of the transcriptional coactivator Yap1. *Sci. Signal* **4**, ra33 (2011). 21610251 10.1126/scisignal.2001823 PMC3366274

[7] Zanconato, F. et al. Genome-wide association between YAP/TAZ/TEAD and AP-1 at enhancers drives oncogenic growth. *Nat. Cell Biol.* **17**, 1218–1227 (2015). 26258633 10.1038/ncb3216 PMC6186417

[8] Sayedyahossein, S., Thines, L. & Sacks, D. B. Ca(2+) signaling and the Hippo pathway: intersections in cellular regulation. *Cell Signal* **110**, 110846 (2023). 37549859 10.1016/j.cellsig.2023.110846 PMC10529277

[9] Wada, K., Itoga, K., Okano, T., Yonemura, S. & Sasaki, H. Hippo pathway regulation by cell morphology and stress fibers. *Development* **138**, 3907–3914 (2011). 21831922 10.1242/dev.070987

[10] Zhao, B. et al. TEAD mediates YAP-dependent gene induction and growth control. *Genes Dev.* **22**, 1962–1971 (2008). 18579750 10.1101/gad.1664408 PMC2492741

[11] Zhang, W. & Liu, H. T. MAPK signal pathways in the regulation of cell proliferation in mammalian cells. *Cell Res.* **12**, 9–18 (2002). 11942415 10.1038/sj.cr.7290105

[12] Therrien, M. et al. KSR, a novel protein kinase required for RAS signal transduction. *Cell* **83**, 879–888 (1995). 8521512 10.1016/0092-8674(95)90204-x

[13] Brown, M. D. & Sacks, D. B. Protein scaffolds in MAP kinase signalling. *Cell Signal* **21**, 462–469 (2009). 19091303 10.1016/j.cellsig.2008.11.013 PMC2668224

[14] Germino, E. A. et al. Homozygous KSR1 deletion attenuates morbidity but does not prevent tumor development in a mouse model of RAS-driven pancreatic cancer. *PLoS One* **13**, e0194998 (2018). 29596465 10.1371/journal.pone.0194998 PMC5875795

[15] Paniagua, G. et al. KSR induces RAS-independent MAPK pathway activation and modulates the efficacy of KRAS inhibitors. *Mol. Oncol.* **16**, 3066–3081 (2022). 35313064 10.1002/1878-0261.13213 PMC9441002

[16] Parvathaneni, S., Li, Z. & Sacks, D. B. Calmodulin influences MAPK signaling by binding KSR1. *J. Biol. Chem.* **296**, 100577 (2021). 33766558 10.1016/j.jbc.2021.100577 PMC8079274

[17] Martin-Vega, A. et al. Scaffold coupling: ERK activation by trans-phosphorylation across different scaffold protein species. *Sci. Adv.* **9**, eadd7969 (2023). 36791195 10.1126/sciadv.add7969 PMC9931222

[18] Jagemann, L. R. et al. The functional interaction of 14-3-3 proteins with the ERK1/2 scaffold KSR1 occurs in an isoform-specific manner. *J. Biol. Chem.* **283**, 17450–17462 (2008). 18426801 10.1074/jbc.M709185200

[19] Bell, B., Xing, H., Yan, K., Gautam, N. & Muslin, A. J. KSR-1 binds to G-protein betagamma subunits and inhibits beta gamma-induced mitogen-activated protein kinase activation. *J. Biol. Chem.* **274**, 7982–7986 (1999). 10075696 10.1074/jbc.274.12.7982

[20] Stewart, S. et al. Kinase suppressor of Ras forms a multiprotein signaling complex and modulates MEK localization. *Mol. Cell Biol.* **19**, 5523–5534 (1999). 10409742 10.1128/mcb.19.8.5523 PMC84397

[21] Muller, J., Ory, S., Copeland, T., Piwnica-Worms, H. & Morrison, D. K. C-TAK1 regulates Ras signaling by phosphorylating the MAPK scaffold, KSR1. *Mol. Cell* **8**, 983–993 (2001). 11741534 10.1016/s1097-2765(01)00383-5

[22] Roy, M., Li, Z. & Sacks, D. B. IQGAP1 is a scaffold for mitogen-activated protein kinase signaling. *Mol. Cell Biol.* **25**, 7940–7952 (2005). 16135787 10.1128/MCB.25.18.7940-7952.2005 PMC1234344

[23] Sayedyahossein, S., Li, Z., Hedman, A. C., Morgan, C. J. & Sacks, D. B. IQGAP1 binds to yes-associated protein (yap) and modulates its transcriptional activity. *J. Biol. Chem.* **291**, 19261–19273 (2016). 27440047 10.1074/jbc.M116.732529 PMC5016668

[24] Quinn, N. P. et al. IQGAP1 is a scaffold of the core proteins of the Hippo pathway and negatively regulates the pro-apoptotic signal mediated by this pathway. *Cells* **10**, 478 (2021). 10.3390/cells10020478 PMC7926663 33672268

[25] Thines, L., Roushar, F. J., Hedman, A. C. & Sacks, D. B. The IQGAP scaffolds: critical nodes bridging receptor activation to cellular signaling. *J. Cell Biol.* **222**, 10.1083/jcb.202205062 (2023). 10.1083/jcb.202205062 PMC10120595 37071417

[26] Weingartner, K. A., Tran, T., Tripp, K. W. & Kavran, J. M. Dimerization and autophosphorylation of the MST family of kinases are controlled by the same set of residues. *Biochem J.* **480**, 1165–1182 (2023). 37459121 10.1042/BCJ20230067 PMC10500444

[27] Jumper, J. et al. Highly accurate protein structure prediction with alphaFold. *Nature* **596**, 583–589 (2021). 34265844 10.1038/s41586-021-03819-2 PMC8371605

[28] Bae, S. J. & Luo, X. Activation mechanisms of the Hippo kinase signaling cascade. *Biosci. Rep.* **38**, BSR20171469 (2018). 10.1042/BSR20171469 PMC6131212 30038061

[29] Plouffe, S. W. et al. Characterization of Hippo pathway components by gene inactivation. *Mol. Cell* **64**, 993–1008 (2016). 27912098 10.1016/j.molcel.2016.10.034 PMC5137798

[30] Brown, M. D. & Sacks, D. B. Compartmentalized MAPK pathways. In *Handb Exp Pharmacol*, (Eds. Scott J. D. & Klussmann, E.) vol. 186, pp. 205–235, (Springer, 2008). 10.1007/978-3-540-72843-6_9 18491054

[31] Zhao, B. et al. Inactivation of YAP oncoprotein by the Hippo pathway is involved in cell contact inhibition and tissue growth control. *Genes Dev.* **21**, 2747–2761 (2007). 17974916 10.1101/gad.1602907 PMC2045129

[32] Kim, M. et al. Expression of kinase suppressor of Ras1 enhances cisplatin-induced extracellular signal-regulated kinase activation and cisplatin sensitivity. *Cancer Res*. **65**, 3986–3992 (2005). 15899786 10.1158/0008-5472.CAN-03-2334

[33] Masuda, M., Nakagawa, R. & Kondo, T. Harnessing the potential of reverse-phase protein array technology: Advancing precision oncology strategies. *Cancer Sci.* **115**, 1378–1387 (2024). 38409909 10.1111/cas.16123 PMC11093203

[34] Borzou, P., Ghaisari, J., Izadi, I., Eshraghi, Y. & Gheisari, Y. A novel strategy for dynamic modeling of genome-scale interaction networks. *Bioinformatics* **39**, 10.1093/bioinformatics/btad079 (2023). 10.1093/bioinformatics/btad079 PMC9969830 36825834

[35] Turner, C. E., Glenney, J. R. Jr. & Burridge, K. Paxillin: a new vinculin-binding protein present in focal adhesions. *J. Cell Biol.* **111**, 1059–1068 (1990). 2118142 10.1083/jcb.111.3.1059 PMC2116264

[36] Das, A., Fischer, R. S., Pan, D. & Waterman, C. M. YAP nuclear localization in the absence of cell-cell contact is mediated by a filamentous actin-dependent, myosin II- and phospho-yap-independent pathway during extracellular matrix mechanosensing. *J. Biol. Chem.* **291**, 6096–6110 (2016). 26757814 10.1074/jbc.M115.708313 PMC4813550

[37] Sansores-Garcia, L. et al. Modulating F-actin organization induces organ growth by affecting the Hippo pathway. *EMBO J.* **30**, 2325–2335 (2011). 21556047 10.1038/emboj.2011.157 PMC3116287

[38] Tkach, V., Bock, E. & Berezin, V. The role of RhoA in the regulation of cell morphology and motility. *Cell Motil. Cytoskelet.* **61**, 21–33 (2005). 10.1002/cm.20062 15776463

[39] Liu, Z. et al. RhoA/ROCK-YAP/TAZ axis regulates the fibrotic activity in dexamethasone-treated human trabecular meshwork cells. *Front. Mol. Biosci.* **8**, 728932 (2021). 34552960 10.3389/fmolb.2021.728932 PMC8450533

[40] Flatau, G. et al. Toxin-induced activation of the G protein p21 Rho by deamidation of glutamine. *Nature* **387**, 729–733 (1997). 9192901 10.1038/42743

[41] Bade, N. D., Kamien, R. D., Assoian, R. K. & Stebe, K. J. Curvature and Rho activation differentially control the alignment of cells and stress fibers. *Sci. Adv.* **3**, e1700150 (2017). 28913421 10.1126/sciadv.1700150 PMC5587136

[42] Kolch, W. Coordinating ERK/MAPK signalling through scaffolds and inhibitors. *Nat. Rev. Mol. Cell Biol.* **6**, 827–837 (2005). 16227978 10.1038/nrm1743

[43] Hao, Y., Chun, A., Cheung, K., Rashidi, B. & Yang, X. Tumor suppressor LATS1 is a negative regulator of oncogene YAP. *J. Biol. Chem.* **283**, 5496–5509 (2008). 18158288 10.1074/jbc.M709037200

[44] Maglic, D. et al. YAP-TEAD signaling promotes basal cell carcinoma development via a c-JUN/AP1 axis. *EMBO J.* **37**, e98642 (2018). 10.15252/embj.201798642 PMC6120663 30037824

[45] Croci, O. et al. Transcriptional integration of mitogenic and mechanical signals by Myc and YAP. *Genes Dev.* **31**, 2017–2022 (2017). 29141911 10.1101/gad.301184.117 PMC5733494

[46] Zhou, L. et al. The scaffold protein KSR1, a novel therapeutic target for the treatment of Merlin-deficient tumors. *Oncogene* **35**, 3443–3453 (2016). 26549023 10.1038/onc.2015.404 PMC4861249

[47] Li, W. et al. Merlin/NF2 loss-driven tumorigenesis linked to CRL4(DCAF1)-mediated inhibition of the hippo pathway kinases Lats1 and 2 in the nucleus. *Cancer Cell* **26**, 48–60 (2014). 25026211 10.1016/j.ccr.2014.05.001 PMC4126592

[48] Mannion, A. J. et al. Regulation of YAP promotor accessibility in endothelial mechanotransduction. *Arterioscler. Thromb. Vasc. Biol.* **44**, 666–689 (2024). 38299356 10.1161/ATVBAHA.123.320300 PMC10880945

[49] Brennan, J. A., Volle, D. J., Chaika, O. V. & Lewis, R. E. Phosphorylation regulates the nucleocytoplasmic distribution of kinase suppressor of Ras. *J. Biol. Chem.* **277**, 5369–5377 (2002). 11741955 10.1074/jbc.M109875200

[50] Rao, C. et al. KSR1- and ERK-dependent translational regulation of the epithelial-to-mesenchymal transition. *Elife* **10**, e66608 (2021). 10.7554/eLife.66608 PMC8195604 33970103

[51] Park, J. et al. Switch-like enhancement of epithelial-mesenchymal transition by YAP through feedback regulation of WT1 and Rho-family GTPases. *Nat. Commun.* **10**, 2797 (2019). 31243273 10.1038/s41467-019-10729-5 PMC6594963

[52] Xia, H. et al. EGFR-PI3K-PDK1 pathway regulates YAP signaling in hepatocellular carcinoma: the mechanism and its implications in targeted therapy. *Cell Death Dis.* **9**, 269 (2018). 29449645 10.1038/s41419-018-0302-x PMC5833379

[53] Birkenfeld, J. et al. GEF-H1 modulates localized RhoA activation during cytokinesis under the control of mitotic kinases. *Dev. Cell* **12**, 699–712 (2007). 17488622 10.1016/j.devcel.2007.03.014 PMC1965589

[54] Cullis, J. et al. The RhoGEF GEF-H1 is required for oncogenic RAS signaling via KSR-1. *Cancer Cell* **25**, 181–195 (2014). 24525234 10.1016/j.ccr.2014.01.025

[55] Nardone, G. et al. YAP regulates cell mechanics by controlling focal adhesion assembly. *Nat. Commun.* **8**, 15321 (2017). 28504269 10.1038/ncomms15321 PMC5440673

[56] Kim, D. H. et al. Actin cap-associated focal adhesions and their distinct role in cellular mechanosensing. *Sci. Rep.* **2**, 555 (2012). 22870384 10.1038/srep00555 PMC3412326

[57] Wang, X. et al. Focal adhesion and actin orientation regulated by cellular geometry determine stem cell differentiation via mechanotransduction. *Acta Biomater.* **182**, 81–92 (2024). 38734287 10.1016/j.actbio.2024.05.017

[58] Liu, Z. et al. Kinase suppressor of RAS 1 (KSR1) maintains the transformed phenotype of BRAFV600E mutant human melanoma cells. *Int. J. Mol. Sci.* **24**. 10.3390/ijms241411821 (2023). 10.3390/ijms241411821 PMC10380721 37511580

[59] Miroshnikova, Y. A. & Wickstrom, S. A. Mechanical forces in nuclear organization. *Cold Spring Harb. Perspect. Biol.* **14**, 10.1101/cshperspect.a039685 (2022). 10.1101/cshperspect.a039685 PMC8725626 34187806

[60] Dahl, K. N., Ribeiro, A. J. & Lammerding, J. Nuclear shape, mechanics, and mechanotransduction. *Circ. Res.* **102**, 1307–1318 (2008). 18535268 10.1161/CIRCRESAHA.108.173989 PMC2717705

[61] Ando, T. et al. EGFR regulates the Hippo pathway by promoting the tyrosine phosphorylation of MOB1. *Commun. Biol.* **4**, 1237 (2021). 34725466 10.1038/s42003-021-02744-4 PMC8560880

[62] Yu, W., Fantl, W. J., Harrowe, G. & Williams, L. T. Regulation of the MAP kinase pathway by mammalian Ksr through direct interaction with MEK and ERK. *Curr. Biol.* **8**, 56–64 (1998). 9427629 10.1016/s0960-9822(98)70020-x

[63] Fan, R., Kim, N. G. & Gumbiner, B. M. Regulation of Hippo pathway by mitogenic growth factors via phosphoinositide 3-kinase and phosphoinositide-dependent kinase-1. *Proc. Natl. Acad. Sci. USA* **110**, 2569–2574 (2013). 23359693 10.1073/pnas.1216462110 PMC3574943

[64] Ren, J. G., Li, Z. & Sacks, D. B. IQGAP1 modulates activation of B-Raf. *Proc. Natl. Acad. Sci. USA* **104**, 10465–10469 (2007). 17563371 10.1073/pnas.0611308104 PMC1965536

[65] Kortum, R. L. et al. The molecular scaffold kinase suppressor of Ras 1 (KSR1) regulates adipogenesis. *Mol. Cell Biol.* **25**, 7592–7604 (2005). 16107706 10.1128/MCB.25.17.7592-7604.2005 PMC1190290

[66] Sayedyahossein, S. et al. Discovery of small molecule inhibitors that effectively disrupt IQGAP1-Cdc42 interaction in breast cancer cells. *Sci. Rep.* **12**, 17372 (2022). 36253497 10.1038/s41598-022-21342-w PMC9576799

[67] Stringer, C., Wang, T., Michaelos, M. & Pachitariu, M. Cellpose: a generalist algorithm for cellular segmentation. *Nat. Methods* **18**, 100–106 (2021). 33318659 10.1038/s41592-020-01018-x

[68] Pachitariu, M. & Stringer, C. Cellpose 2.0: how to train your own model. *Nat. Methods* **19**, 1634–1641 (2022). 36344832 10.1038/s41592-022-01663-4 PMC9718665

[69] Sayedyahossein, S., Hedman, A. C. & Sacks, D. B. Insulin suppresses transcriptional activity of yes-associated protein in insulin target cells. *Mol. Biol. Cell* **31**, 131–141 (2020). 31693448 10.1091/mbc.E19-04-0205 PMC6960410

