# Supplementary material for: KSR1 is a scaffold for the Hippo signaling pathway
Source: Commun Biol. 2025 Dec 1;8:1725. doi: 10.1038/s42003-025-09009-4 (PMC12669712; doi:10.1038/s42003-025-09009-4)

**
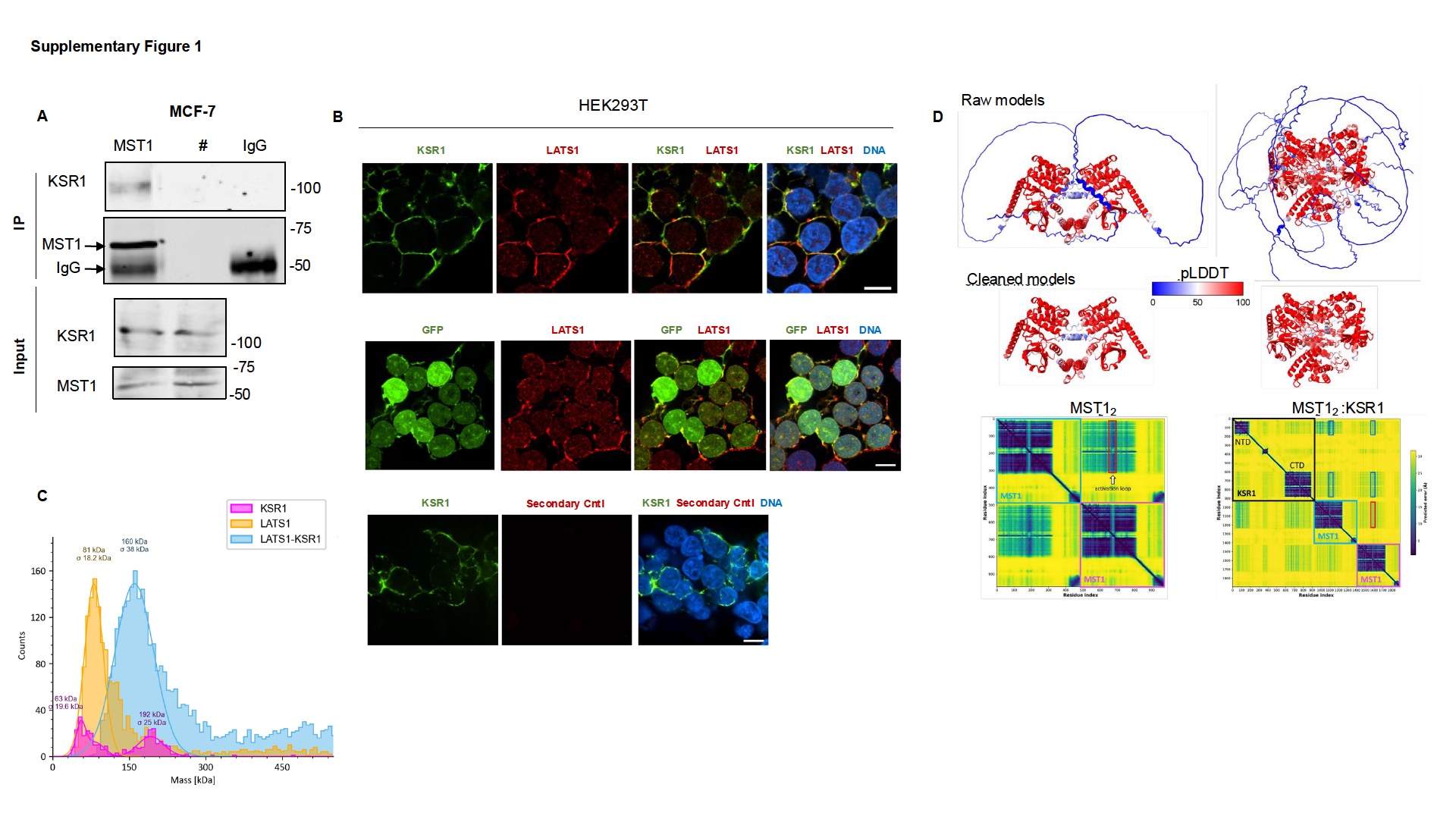
**

**Supplementary Figure Legends**

**Supplementary Figure 1:** **A)** MCF7 cells were lysed and immunoprecipitated (IP) with anti-MST1 antibody.  Rabbit IgG was the negative control. Samples were analyzed by Western blotting. Input represents 5% of the sample volume. # indicates an empty lane. Data are representative of two biological replicates. **B)** HEK293T cells were transfected with either GFP-tagged KSR1 (top panels) or GFP (middle panels) (*green*) as control. Cells were fixed and stained with anti-LATS1 monoclonal antibody (*red)*. DNA was stained with Hoechst (*blue*).  HEK293T cells incubated with only secondary antibodies are controls (bottom *panels*). Data are representative of ≥ 50 cells imaged with a Zeiss LSM880 confocal microscope using a 63× objective lens. Scale bar, 10 μm. **C)** Mass photometry was used to determine the molecular mass distributions of individually purified KSR1 (magenta), LATS1 (orange), and their mixture (blue). KSR1 and LATS1 pure recombinant proteins were diluted to 20 nM in PBS containing 1 mM calcium. The X-axis denotes the molecular masses in kDa and the Y-axis denotes the molecules count. **D)** Top-ranked raw models of MST1_2_ (left column) and MST1_2_:KSR1 (right) complexes generated by AlphaFold2 multimer. The full-length (raw) models (upper panels) were post-processed to obtain (cleaned) models with low- score predicted local distance difference test (pLDDT) confidence score discarded (middle panels); the corresponding pLDDT and predicted aligned error (PAE) matrices are shown (lower panels). The interfaces with the activation loops have higher PAE values than any other regions, both in the MST1 dimer and its complex with KSR1 (red- and blue-contoured rectangular boxes). Lower-ranked AF2 models show similar interfaces, despite some variations in their relative orientations.


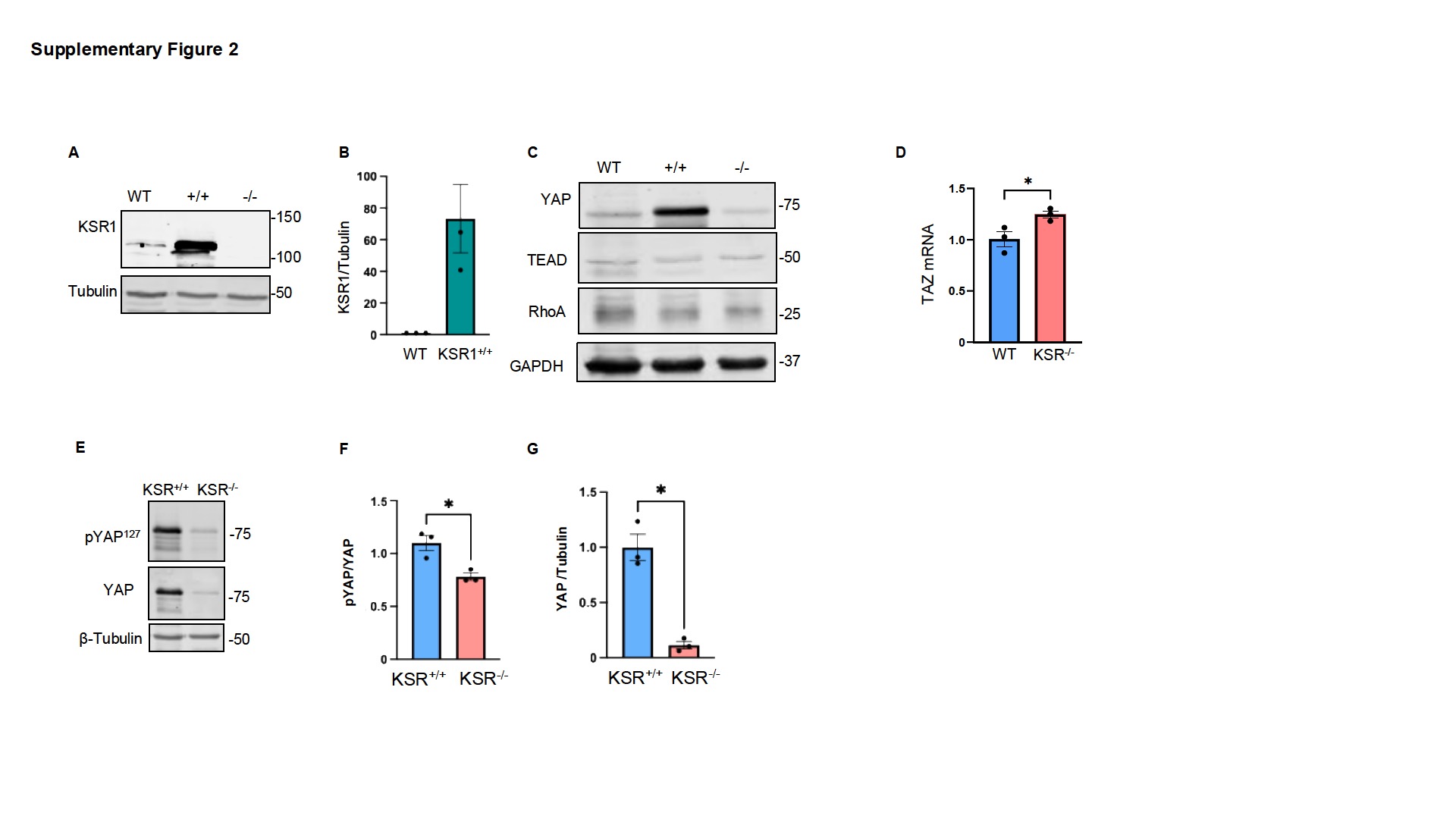


**Supplementary Figure 2: A)** Equal amounts of protein from cell lysates of wild type (WT), KSR1^+/+^ (+/+) and KSR1^-/-^ (-/-) MEFs were analyzed by Western blotting. Blots were probed with antibodies to KSR1 and β-tubulin (loading control). **B)** Bands were quantified and the amount of KSR1 was corrected for β-tubulin in the same sample. Data are expressed as means ± S.E. (error bars), with WT MEFs set as 1 (N = 3). **C)** Equal amounts of protein lysate from MEFs were analyzed by Western blotting performed with the indicated antibodies. Data are representative of three independent experiments. **D)** Total RNA was extracted from WT and KSR1^-/-^ MEFs. TAZ hnRNA was measured by quantitative RT-PCR and corrected for GAPDH hnRNA in the same sample. Data represent the means ± S.E. (error bars) of three independent experiments, each performed in triplicate. Student's *t* test *, *p* <0.05. **E-G)** Equal amounts of protein lysate from KSR1^+/+^ and KSR1^-/-^ MEFs were analyzed by Western blotting, probed with the indicated antibodies. Data are representative of three independent experiments. The bands were quantified and the amount of pYAP to total YAP and total YAP to tubulin in the same sample were calculated (N = 3). Data are expressed as means ± S.E. (error bars).

**
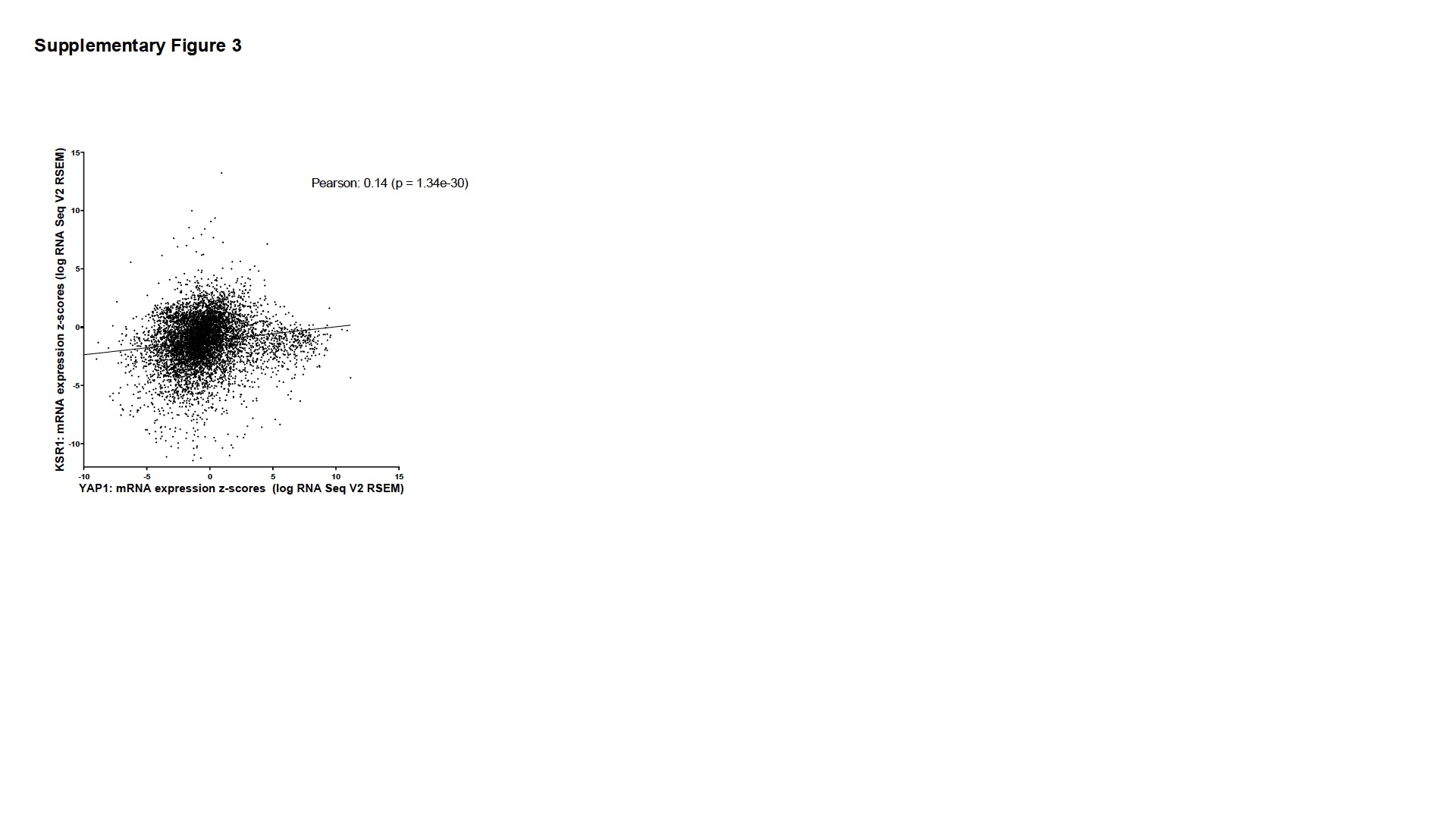
**

**-**

**Supplementary Figure 3:** Correlation between mRNA of KSR1 and YAP in The Cancer Genome Atlas (TCGA) database from all cancer types (10967 biopsy samples, Z-score threshold ± 2).

**
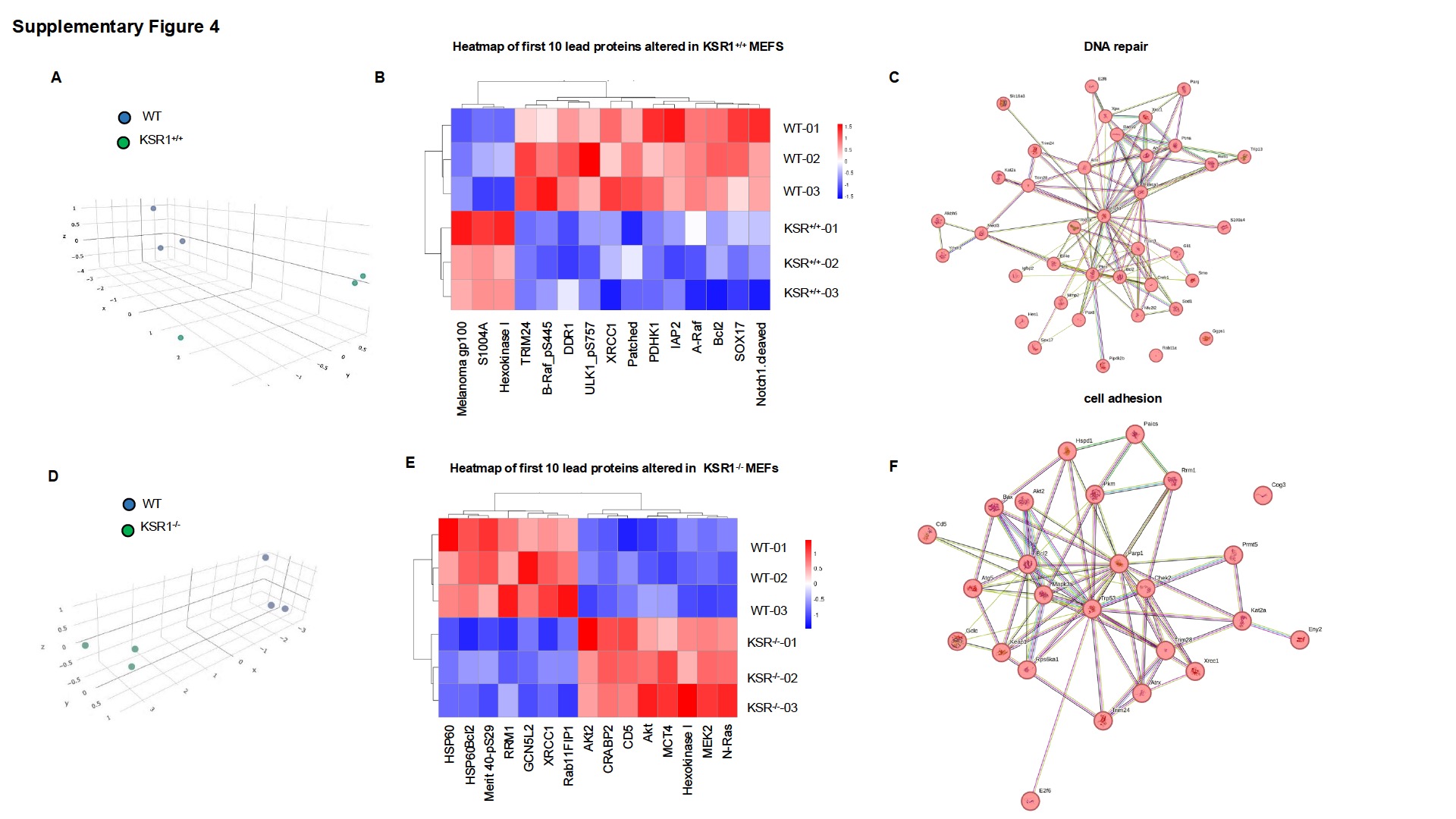
**

**Supplementary Figure 4:** Reverse Phase Protein Array (RPPA) analysis of WT, KSR1^+/+^ and KSR1^-/-^ MEFs. Protein lysates were isolated from WT (*N*=3), KSR1^+/+^ (*N*=3) and KSR1^-/-^ (*N*=3) MEFs for RPPA analysis. **A and D)** Principal component analysis (PCA) shows each cell type cluster. **B and E)** Heat maps showing the 10 highest ranked, differentially expressed proteins between WT (WT 01–03) and KSR1^+/+^ (KSR^+/+^ 01–03) MEFs in (B) and WT (WT 01–03) and KSR1^-/-^ (KSR^-/-^ 01–03) MEFs in (E). Individual proteins listed have a fold change of at least ±2 and *p* < 0.05. **C and F)** STRING plots were generated using the 30 highest ranked differentially expressed proteins showing ±2-fold expression changes. K-means clustering was applied to categorize the output into three distinct clusters.


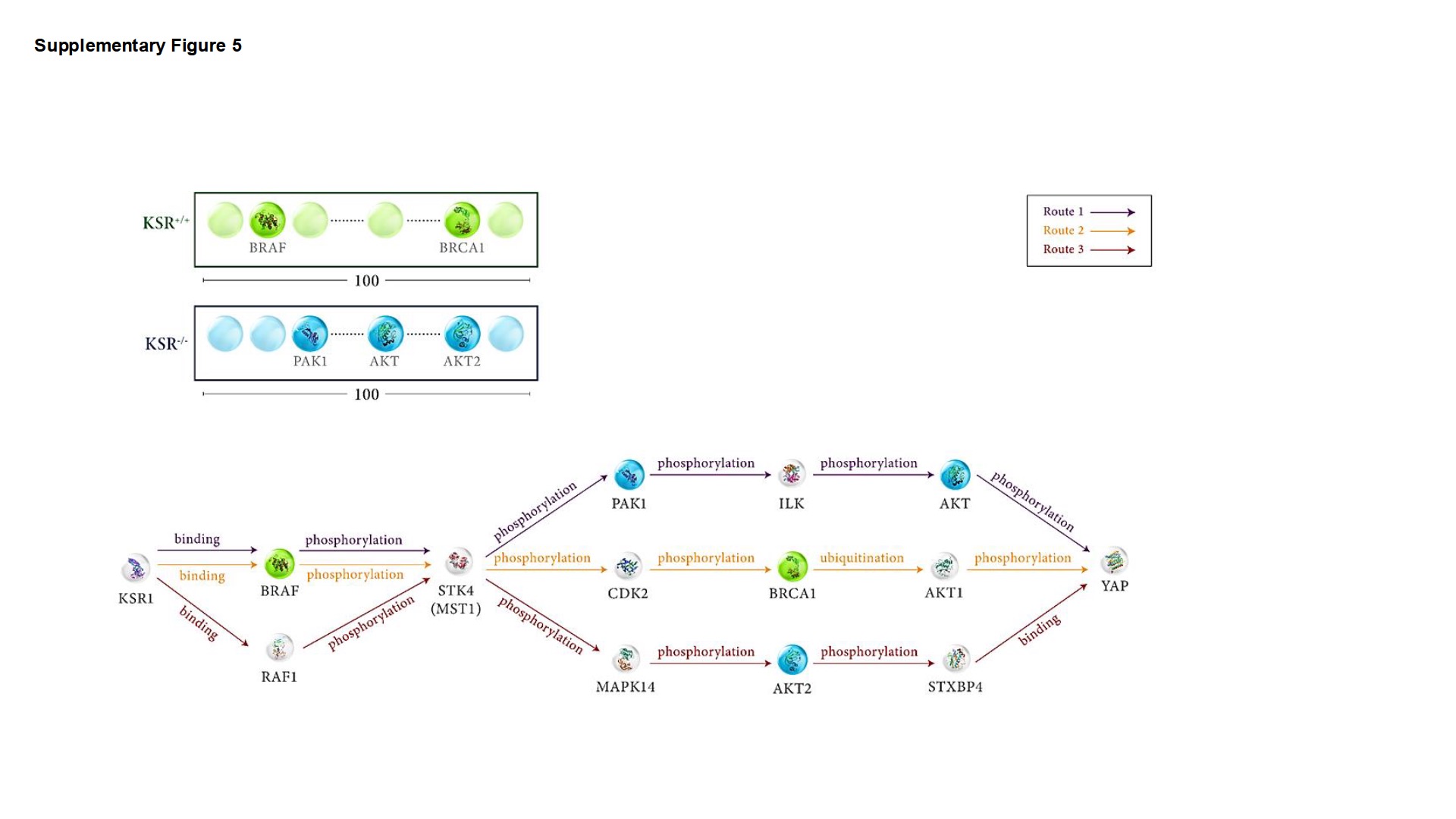


**Supplementary Figure 5:** We used a MATLAB toolbox for exploring and modeling PPI networks (SPADAN) to identify potential routes from KSR1 to YAP1.The results yield 11 possible routes with three mediators. Increasing the number of mediators to four and five resulted in 75 and 968 possible routes, respectively. The identified routes were scored for assessment, providing insights from the alignment with the RPPA results presented in Supplementary Figure 4. The score of each route includes: 1. The count of nodes within the route that correspond to the top genes selected from RPPA. 2. The number of null genes that could be directly linked by a Protein-Protein Interaction (PPI) edge with at least one of the route nodes. 3. The number of genes accessible with a single mediator, originating from at least one of the route nodes.

**
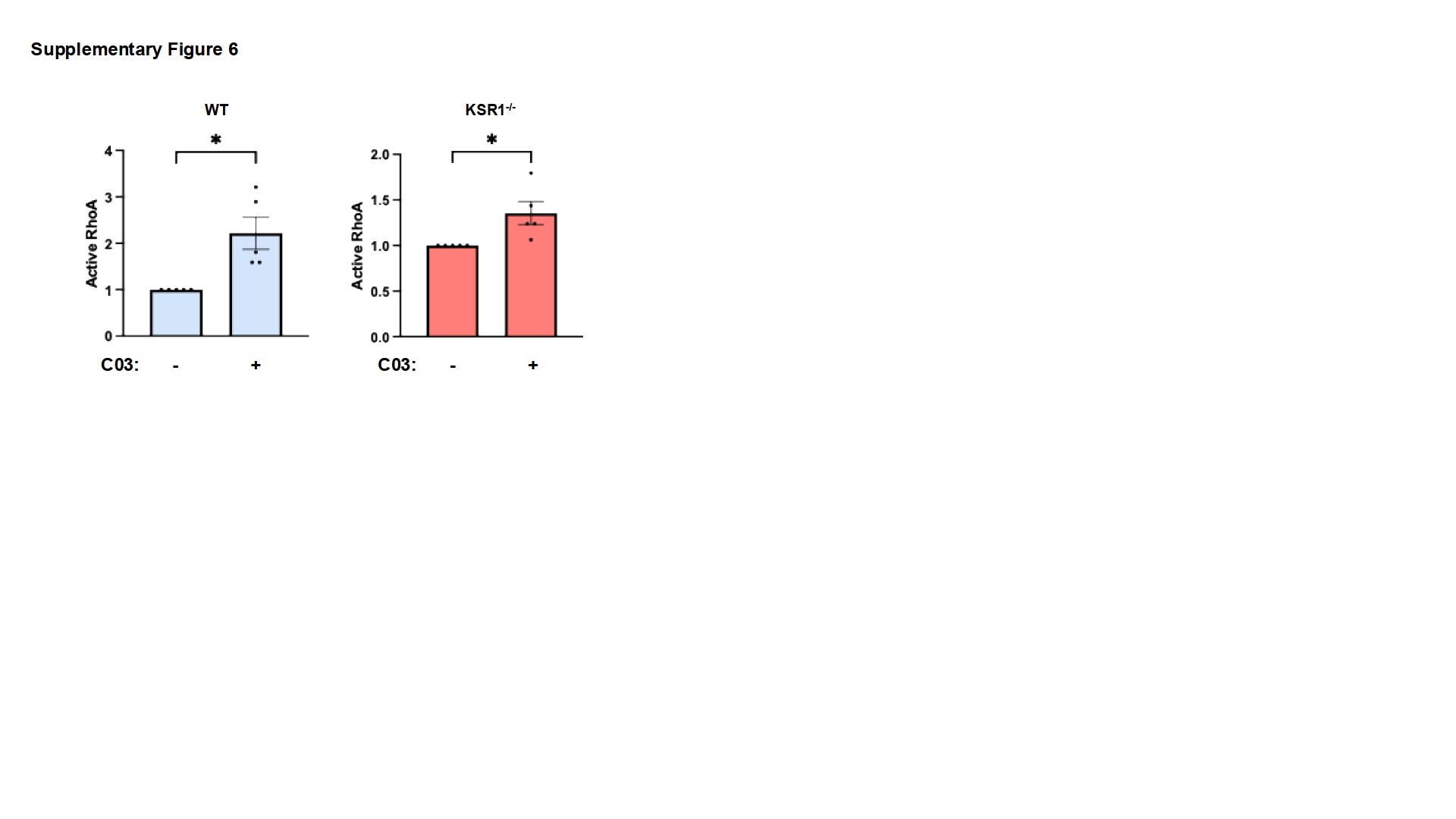
**

**Supplementary Figure 6:** WT and KSR1^-/-^ MEFs were starved of serum for 6 h, then incubated with either vehicle (ddH2O) (-) or the RhoA activator CN03 (+) for 4 h. RhoA-GTP (active RhoA) was quantified using the G-LISA assay. Data represent means ± S.E., *N=3, n=3*, * *p* < 0.05.

**Full blots of the figures**


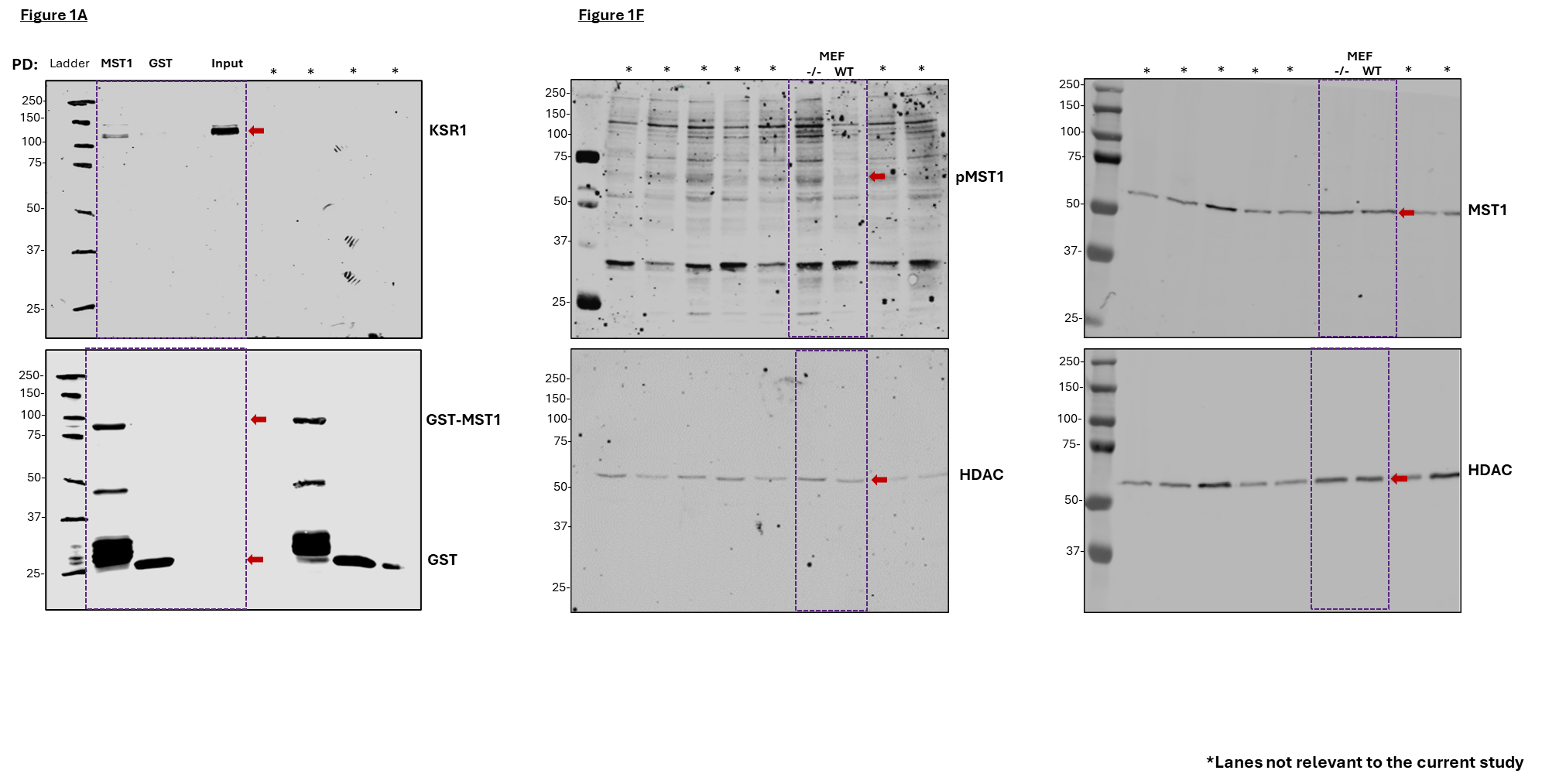


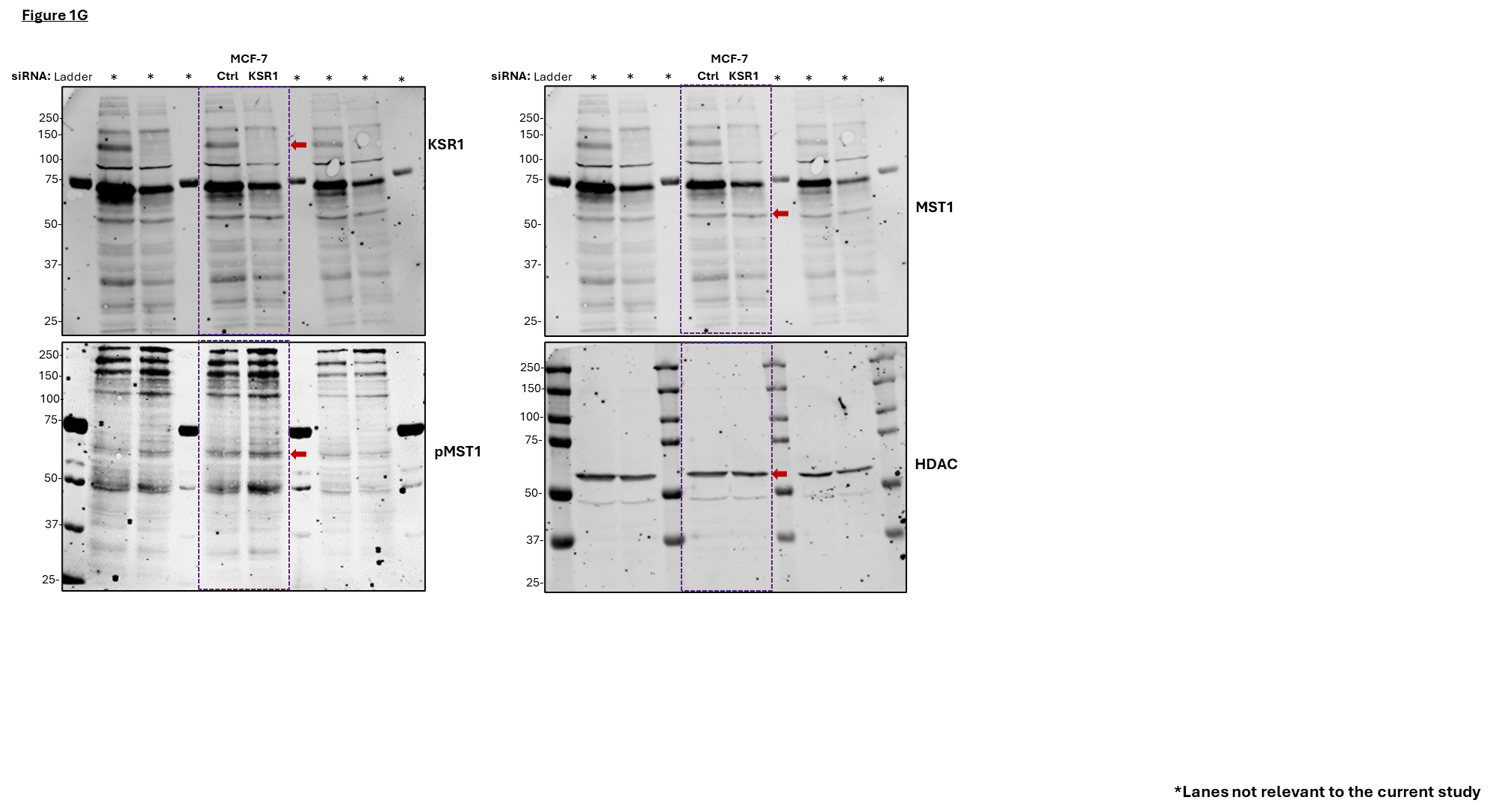


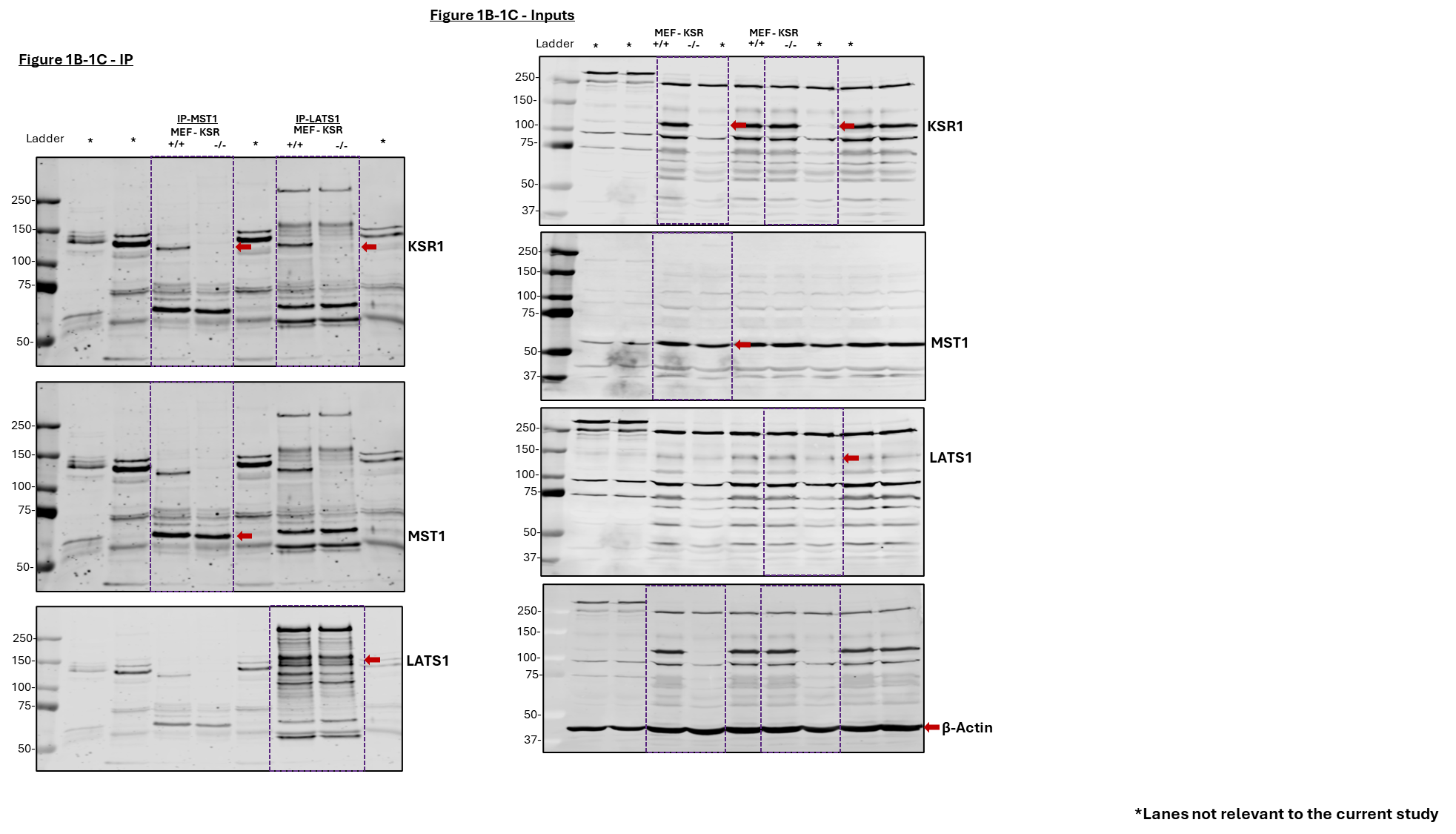


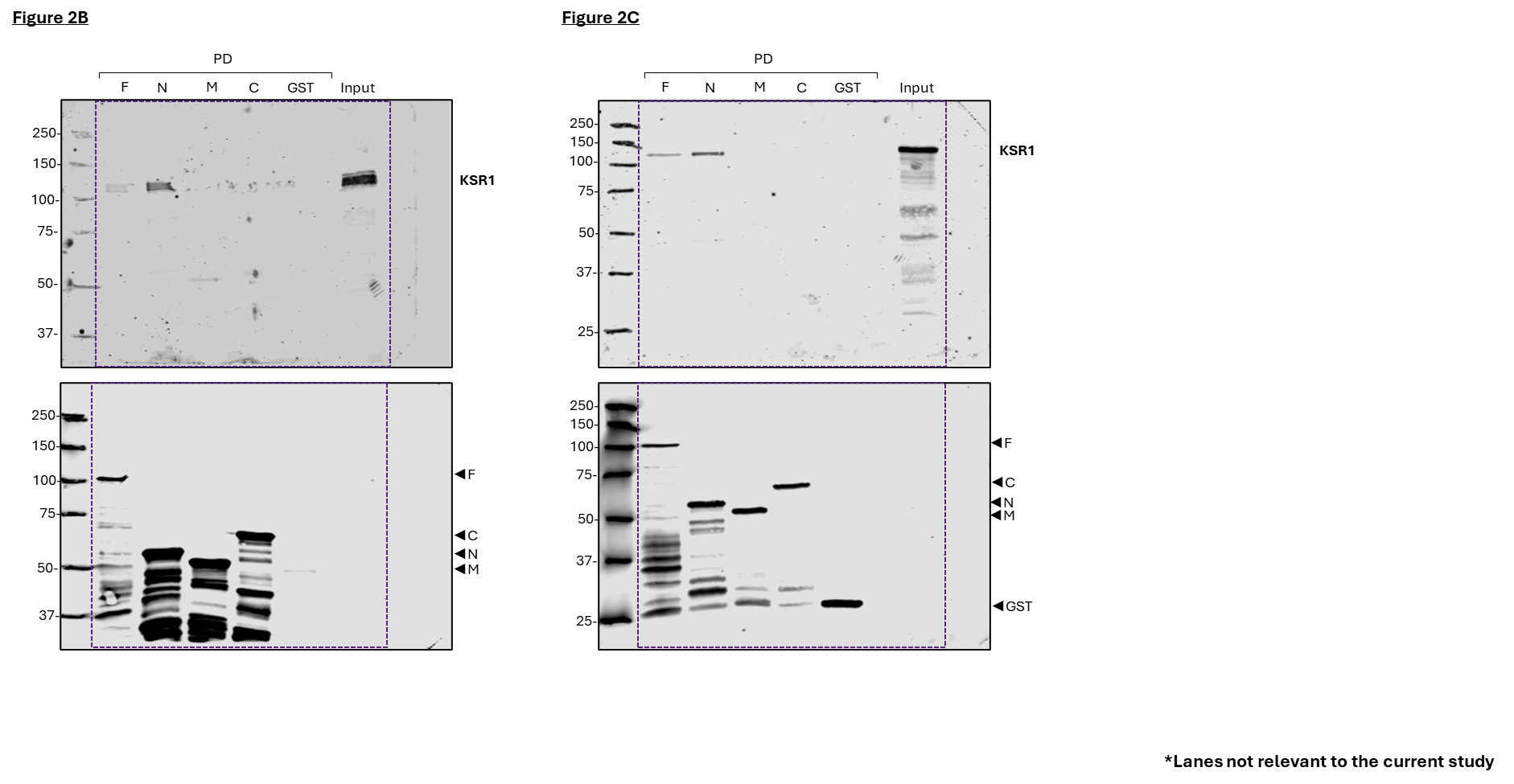


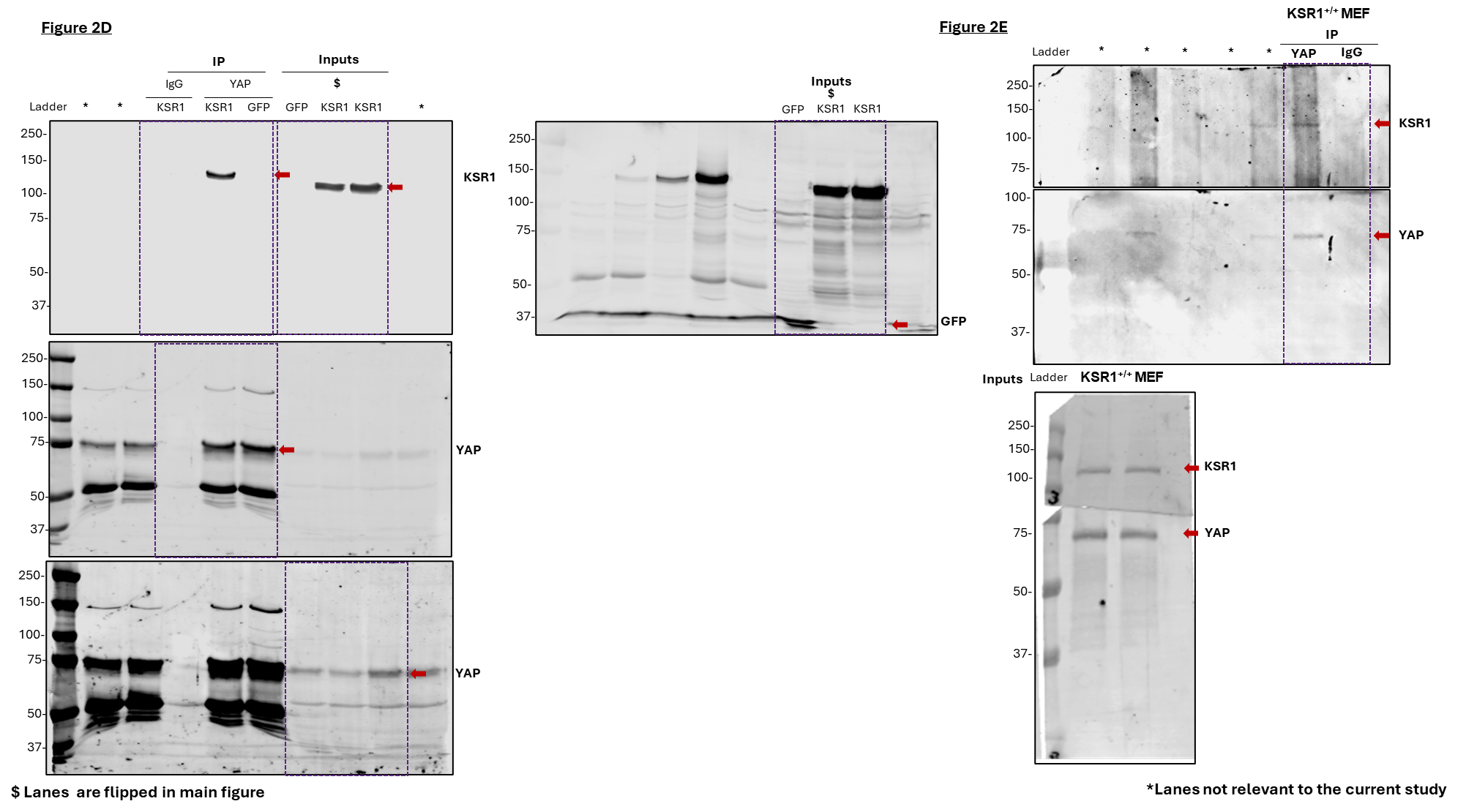


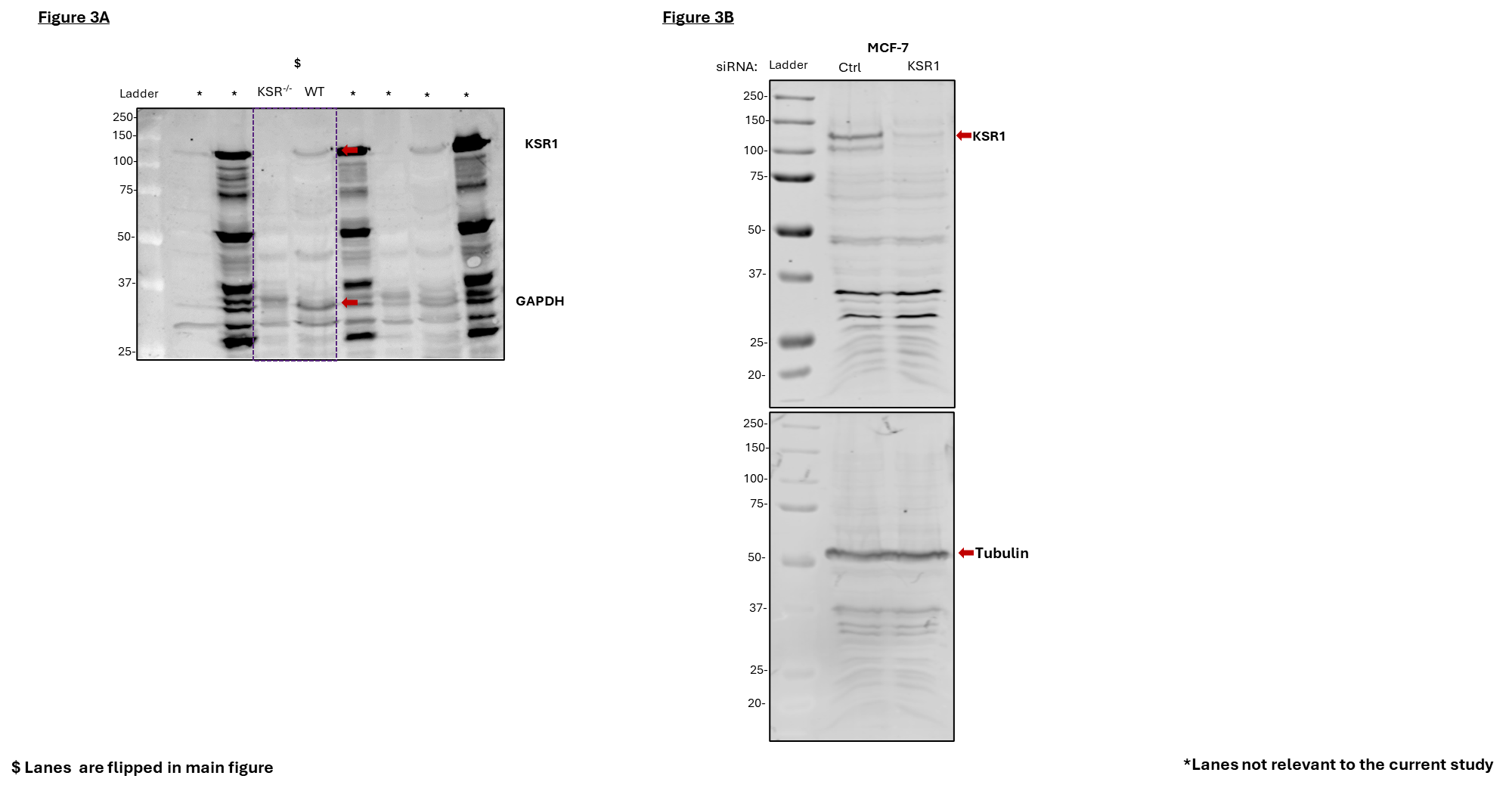


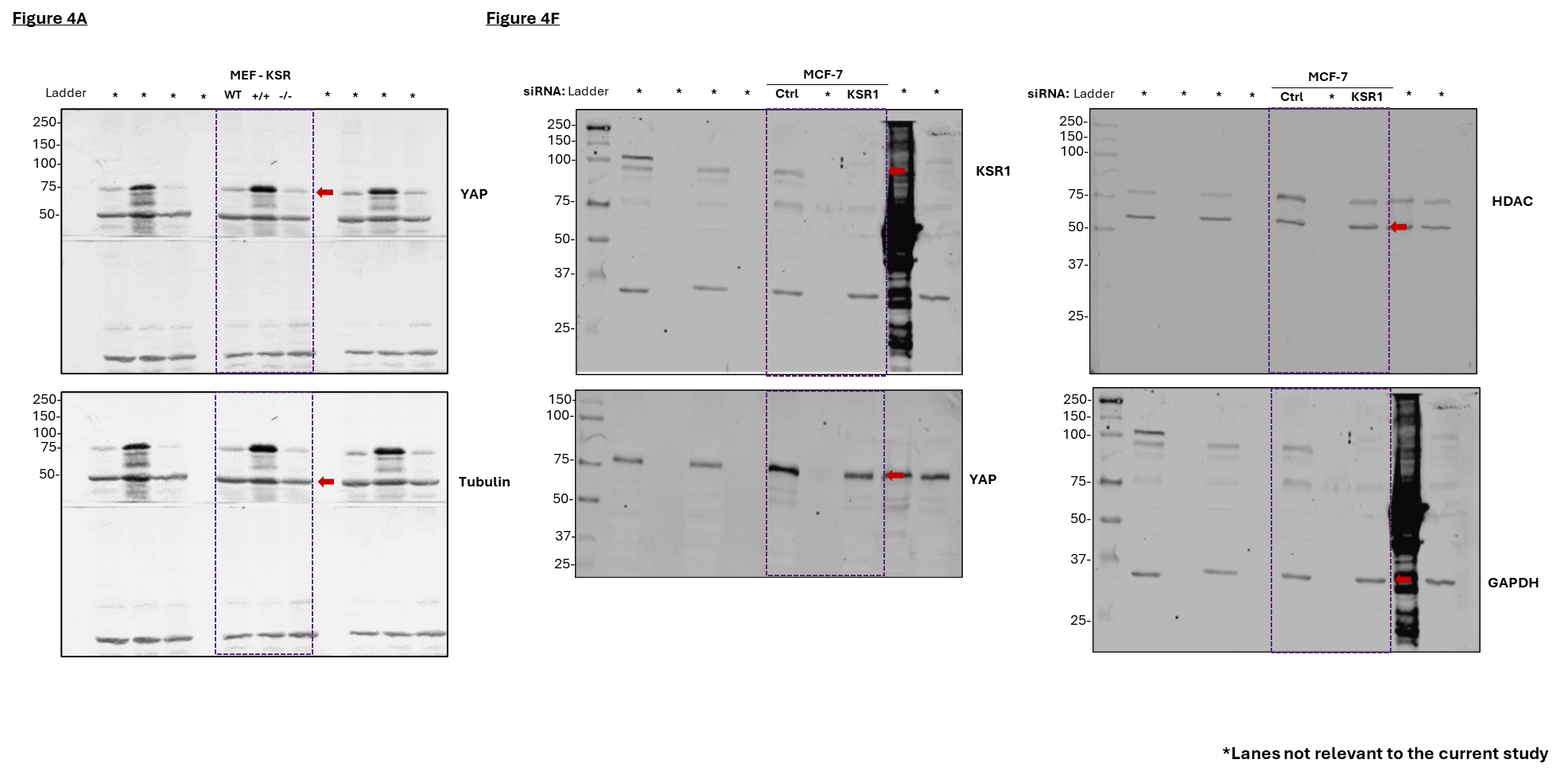


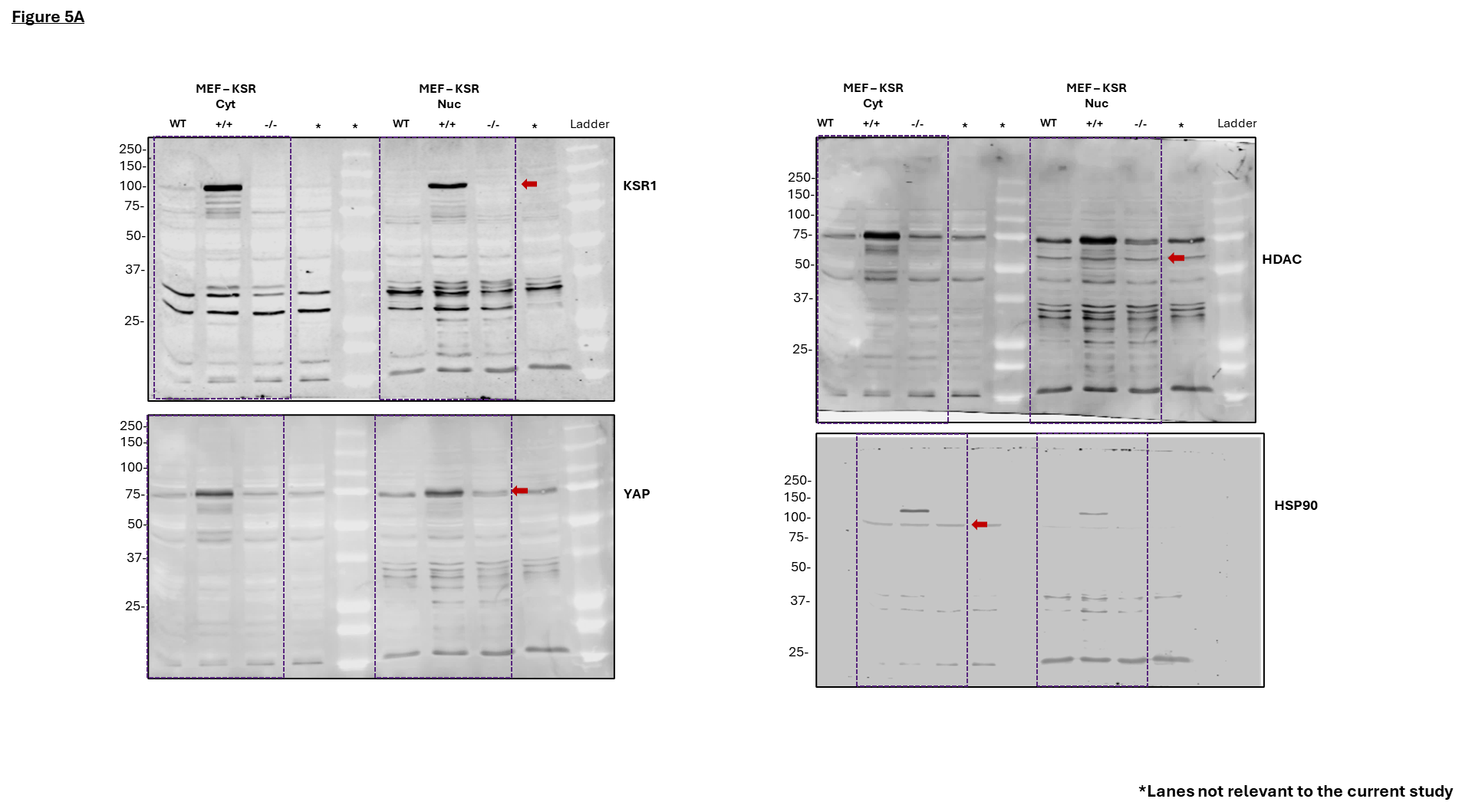


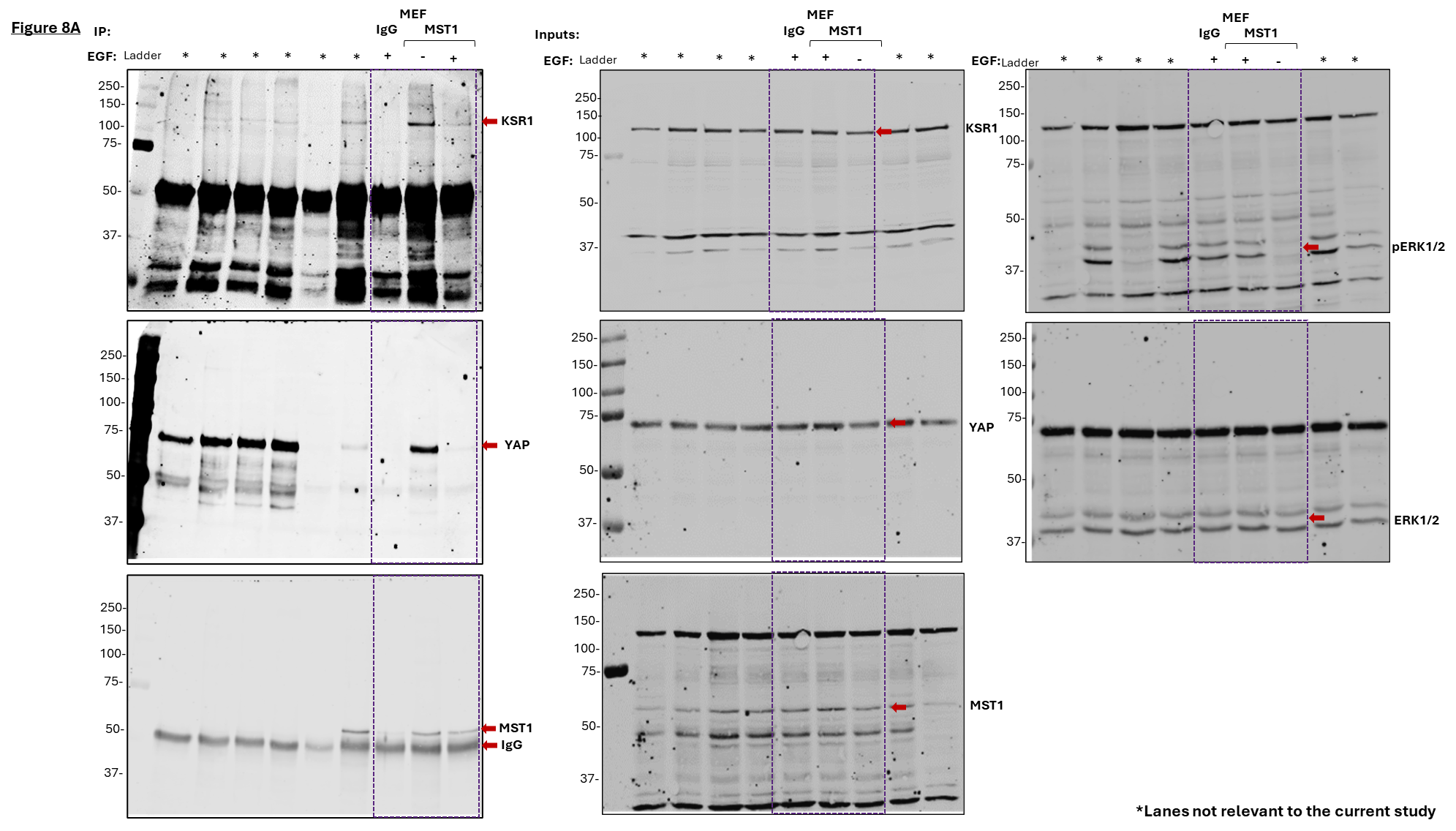


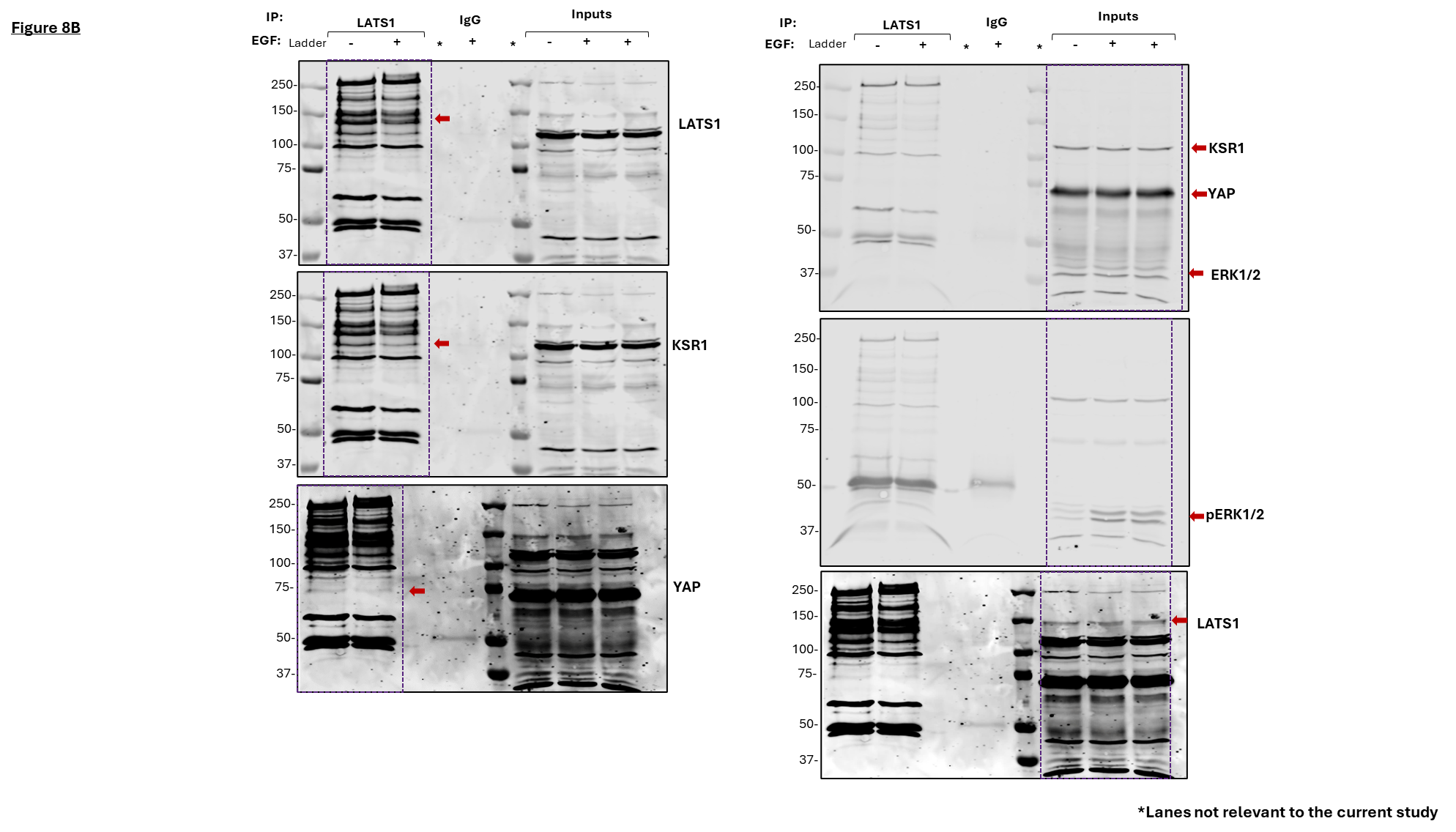


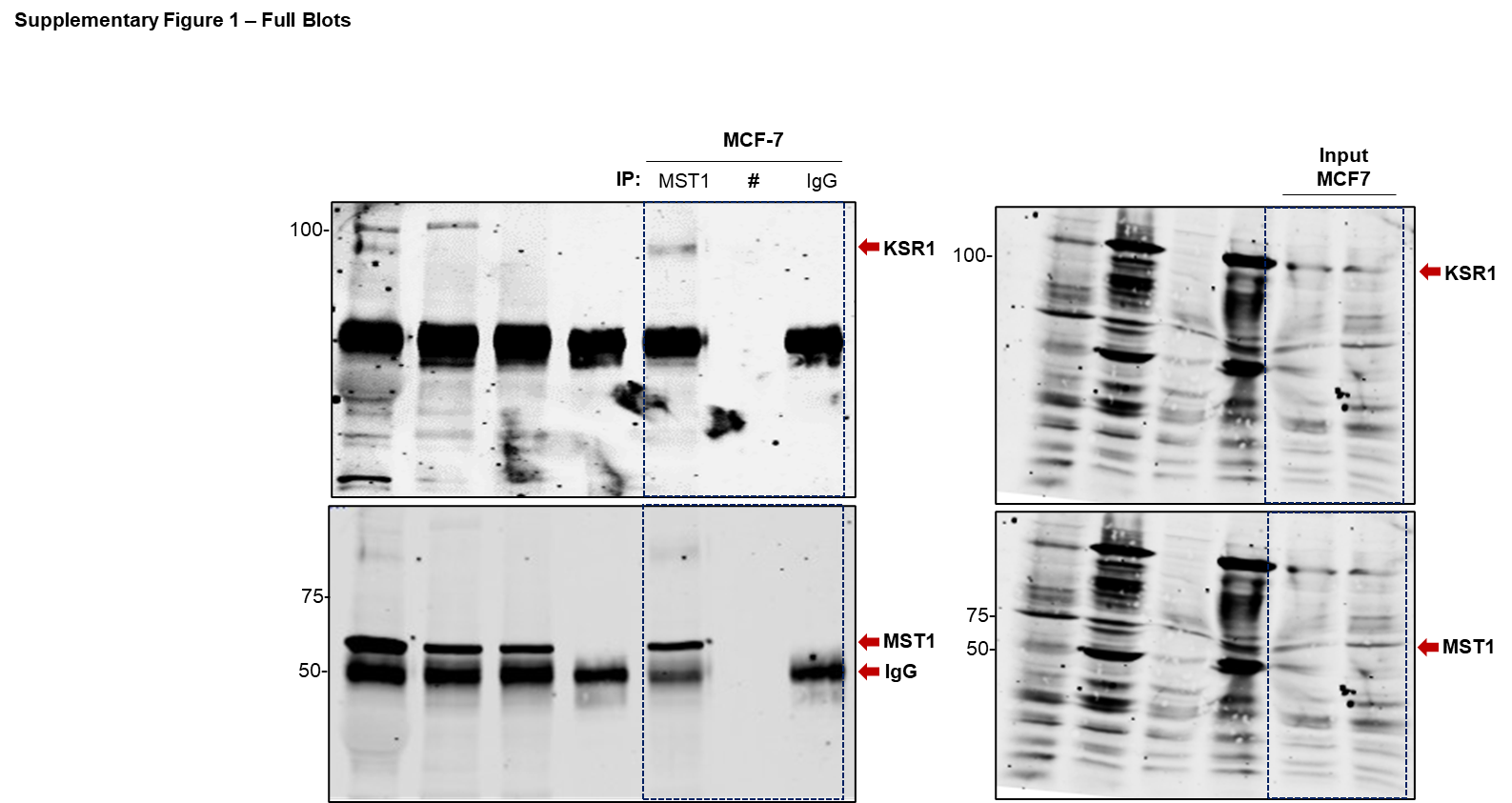


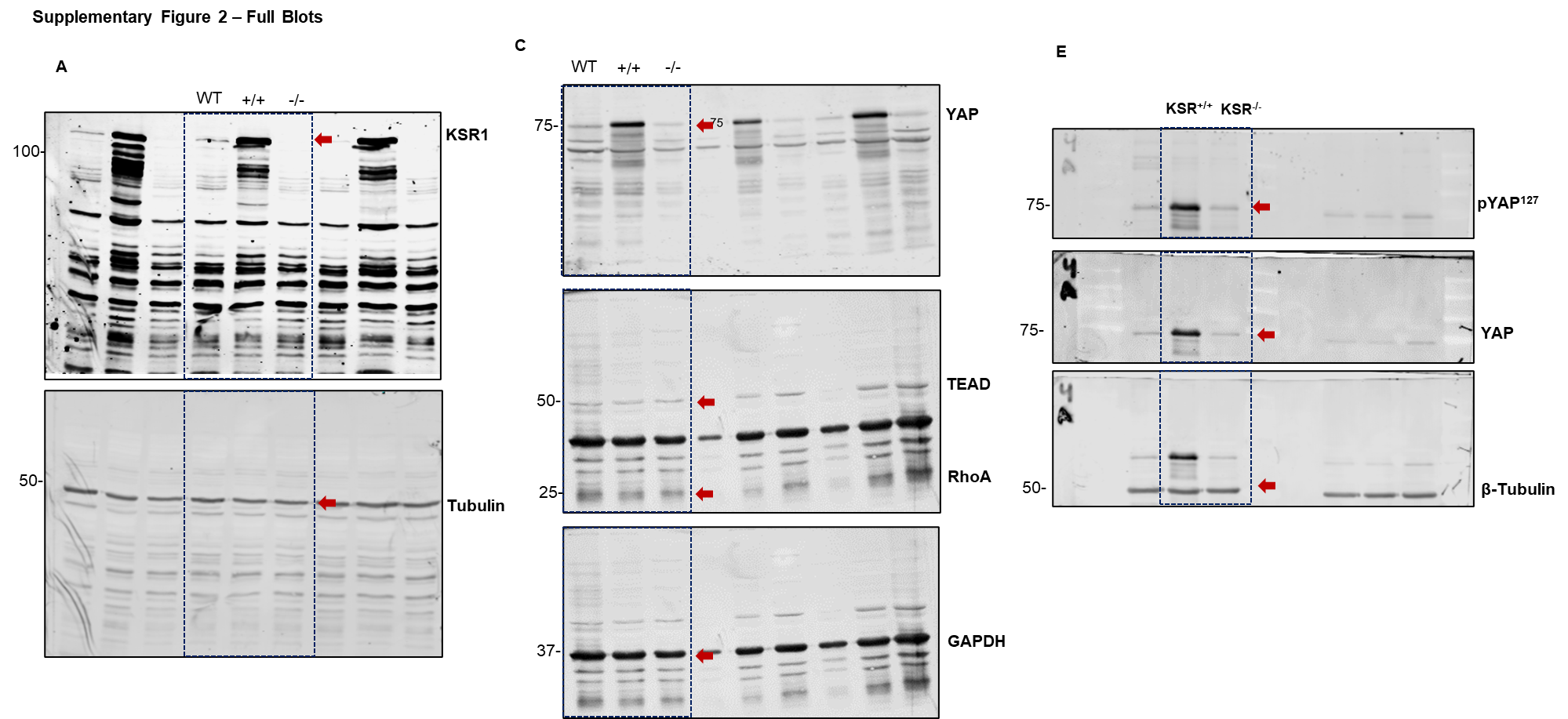

Supplement: Supplementary file 1 — Supplementary information [file 42003_2025_9009_MOESM1_ESM.docx]
