# Supplementary material for: KSR1 is a scaffold for the Hippo signaling pathway
Source: Commun Biol. 2025 Dec 1;8:1725. doi: 10.1038/s42003-025-09009-4 (PMC12669712; doi:10.1038/s42003-025-09009-4)
Supplement: Supplementary file 2 — Description of Additional Supplementary Materials [file 42003_2025_9009_MOESM2_ESM.pdf]

Description of Additional Supplementary Files

File name: Supplementary Data 1

Description: the numerical source data for graphs and charts
